# Interleukin-8/CXCR2 signaling regulates therapy-induced plasticity and enhances tumorigenicity in glioblastoma

**DOI:** 10.1038/s41419-019-1387-6

**Published:** 2019-03-29

**Authors:** Tanwir Hasan, Seamus P. Caragher, Jack M. Shireman, Cheol H. Park, Fatemeh Atashi, Shivani Baisiwala, Gina Lee, Donna Guo, Jennifer Y. Wang, Mahua Dey, Meijing Wu, Maciej S. Lesniak, Craig M. Horbinski, C. David James, Atique U. Ahmed

**Affiliations:** 1 Department of Neurological Surgery, Feinberg School of Medicine, Northwestern University, Chicago, IL USA; 2 Department of Neurosurgery, Indiana University School of Medicine, Indianapolis, IN 46202 USA; 3 Department of Pathology, Feinberg School of Medicine, Northwestern University, Chicago, IL USA

## Abstract

Emerging evidence reveals enrichment of glioma-initiating cells (GICs) following therapeutic intervention. One factor known to contribute to this enrichment is cellular plasticity—the ability of glioma cells to attain multiple phenotypes. To elucidate the molecular mechanisms governing therapy-induced cellular plasticity, we performed genome-wide chromatin immunoprecipitation sequencing (ChIP-Seq) and gene expression analysis (gene microarray analysis) during treatment with standard of care temozolomide (TMZ) chemotherapy. Analysis revealed significant enhancement of open-chromatin marks in known astrocytic enhancers for interleukin-8 (IL-8) loci as well as elevated expression during anti-glioma chemotherapy. The Cancer Genome Atlas and Ivy Glioblastoma Atlas Project data demonstrated that IL-8 transcript expression is negatively correlated with GBM patient survival (*p* = 0.001) and positively correlated with that of genes associated with the GIC phenotypes, such as KLF4, c-Myc, and HIF2α (*p* < 0.001). Immunohistochemical analysis of patient samples demonstrated elevated IL-8 expression in about 60% of recurrent GBM tumors relative to matched primary tumors and this expression also positively correlates with time to recurrence. Exposure to IL-8 significantly enhanced the self-renewing capacity of PDX GBM (average threefold, *p* < 0.0005**)**, as well as increasing the expression of GIC markers in the CXCR2 population. Furthermore, IL-8 knockdown significantly delayed PDX GBM tumor growth in vivo (*p* < 0.0005). Finally, guided by in silico analysis of TCGA data, we examined the effect of therapy-induced IL-8 expression on the epigenomic landscape of GBM cells and observed increased trimethylation of H3K9 and H3K27. Our results show that autocrine IL-8 alters cellular plasticity and mediates alterations in histone status. These findings suggest that IL-8 signaling participates in regulating GBM adaptation to therapeutic stress and therefore represents a promising target for combination with conventional chemotherapy in order to limit GBM recurrence.

## Introduction

Glioblastoma (GBM) is the most aggressive and prevalent primary brain tumor in adults, with 10,000 new diagnoses each year. Recurrent tumors, with increased invasive and resistance capacities, are an inevitability for GBM patients despite aggressive therapeutic intervention. Glioma-initiating cells (GICs) are considered a key driver of primary tumor development, as well as major contributors to tumor recurrence^1^.

Recent reports demonstrate that differentiated GBM cells undergo cellular and molecular changes to acquire GIC-like states^2,3^. This cellular plasticity dramatically complicates our ability to prevent tumor recurrence in the clinical setting. Our group and others have shown that therapeutic stress and microenvironmental dynamics induce cellular plasticity in GBM, driving the conversion of differentiated GBM cells to GIC states^3–5^. However, the exact mechanisms governing post-therapy GBM plasticity remain unknown. Unraveling the signaling pathways that drive this plasticity will provide key insight for improving treatment of GBM.

Using gene expression and bioinformatic analysis, we identified interleukin-8 (IL-8) as a key player in promoting post-therapy cellular plasticity. IL-8 enhances the self-renewal capacity of patient-derived xenograft (PDX) GBM cells and is elevated in recurrent GBM patient specimens. Reducing levels of IL-8 in murine models significantly improved survival and enhanced the efficacy of Temozolomide chemotherapy. We demonstrate that IL-8/CXCR2 signaling alters the epigenomic landscape in GBM cells, inducing a GIC-like state and increasing the proportion of GICs after treatment. This study highlights IL-8 signaling as a key influence on GBM plasticity and recurrence as well as a potential novel therapeutic target in GBM.

## Materials and methods

### Cell culture

U251 human glioma cell lines were procured from the American Type Culture Collection (Manassas, VA, USA). These cells were cultured in Dulbecco’s Modified Eagle’s Medium (DMEM; HyClone, Thermo Fisher Scientific, San Jose, CA, USA) supplemented with 10% fetal bovine serum (FBS; Atlanta Biologicals, Lawrenceville, GA, USA) and 1% penicillin–streptomycin (P/S) antibiotic mixture (Cellgro; Herdon, VA, USA; Mediatech, Herdon, VA, USA). PDX glioma specimens (GBM43, GBM12, GBM6, GBM5, and GBM39) were obtained from Dr. C. David James at Northwestern University and maintained according to published protocols^6^. Cells were propagated in vivo by injection into the flank of nu/nu athymic nude mice. In vitro experiments with these cells were performed utilizing DMEM supplemented with 1% FBS and 1% P/S antibiotic mixture. All cells were maintained in humidified atmosphere with CO_2_ and temperature carefully kept at 5% and 37 °C, respectively. Dissociations were performed enzymatically using 0.05% trypsin and 2.21 mml/L EDTA solution (Mediatech, Corning, Corning, NY, USA). For experiments, cells were cultured in their appropriate cell culture media treated with temozolomide (TMZ; Schering Plough; stock solution 50 mmol/L in DMSO), Interleukin-8 (IL-8; Peprotech Company, Rocky Hill, NJ, USA), or equimolar DMSO vehicle control. For IL-8 neutralizing antibody experiments, cells were cultured as described above in the presence of an IL-8 neutralizing antibody or an IgG control antibody (R&D Systems, Minneapolis, MN, USA).

### Animals

Athymic nude mice (nu/nu; Charles River, Skokie, IL, USA) were housed according to all Institutional Animal Care and Use Committee (IACUC) guidelines and in compliance with all applicable federal and state statutes governing the use of animals for biomedical research. Briefly, animals were housed in shoebox cages with no more than five mice per cage in a temperature and humidity-controlled room. Food and water were available ad libitum. A strict 12-h light–dark cycle was maintained.

Intracranial implantation of glioblastoma cells was performed as previously published^7^. Briefly, animals received prophylactic injection of buprenex and metacamp via intraperitoneal (i.p.) injection, followed by an i.p. injection of ketamine/xylazine anesthesia mixture (Henry Schien, New York, NY, USA). Sedation was confirmed by foot pinch. Artificial tears were applied to each eye, and the scalp was sterilized repeatedly with betadine and ethanol. The scalp was then bisected using a scalpel to expose the skull. A drill was used to make a small burr hole above the right frontal lobe (~1-mm in diameter). Animals were then placed into the stereotactic rig, and a Hamilton syringe loaded with the cells was brought into the burr hole. The needle point was lowered 3 mm from the dura and injection of 5 µL of cell mixture took place over 1 min. The needle was then raised slightly and left undisturbed for 1 min to ensure proper release of the cell mixture. After this, the syringe was carefully removed. The animal’s head position was maintained, and the skin of the scalp was closed with sutures (Ethicon, Cincinnati, OH, USA). Animals were then placed in fresh cages with circulating heat underneath and monitored for recovery. All instruments were sterilized with a bead sterilizer between animals and all other necessary procedures to maintain a sterile field were performed.

Drug treatments were initiated 7 days after intracranial implantation. Animals received i.p. injections of either TMZ (2.5 mg/kg) or equimolar DMSO. Injections were performed daily for 5 consecutive days.

After injections, animals were monitored daily by a blinded experimenter for signs of sickness, including reduction in body weight, lowered body temperature, lack of grooming, hunched appearance, and behavioral changes. Animals were euthanized when, in the opinion of the blinded experimenter, they would not survive until the next day. Killing of animals was performed according to Northwestern University guidelines. Briefly, animals were placed into CO_2_ chambers and the flow of CO_2_ was initiated; the flow rate did not exceed 2 L CO_2_/min while the animals were conscious. Whole brains were removed and washed in ice-cold phosphate buffer saline (PBS; Corning, Corning, NY, USA). For those brains utilized for FACS analysis, please see Flow Cytometry section of the Materials and methods. For those brains employed for immunohistochemistry analysis, please see the Immunohistochemistry section of the Materials and methods.

### RNA isolation and microarray

After treatment, cells were dissociated with trypsin and washed with PBS. RNA extraction was performed using Qiagen’s RNeasy kit (Qiagen Inc., Germantown, MD, USA) according to the manufacturer’s instructions. Quantification of RNA concentrations was performed using a NanoDrop (Thermo Fisher), and cDNA was synthesized according to established protocols using BioRad’s iScript kit using 1000 ng of the total RNA per sample (BioRad, Hercules, CA, USA). The following cycles were used in a C1000 Thermal Cycler (BioRad) to synthesize cDNA: 5 min at 25 °C, 30 min at 42 °C, 5 min at 85 °C, and then temperature stabilized to 4 °C.

For gene expression analysis, the Illumina HumanHT-12 v3 BeadChip array (Illumina, San Diego, USA) was used. GBM43 PDX GBM was treated with TMZ (50 μM) for 8 days. Cells were harvested and mRNA was isolated as described above. Samples preparation and mRNA array hybridization was performed according to the manufacturer’s guidelines. Illumina Bead Array Reader was used to read the array and the Illumina’s GenomeStudio software, as well as the Gene Expression Module was utilized for data analysis. For quality control, samples with less than 6000 significant detection probes (detection *p-*value < 0.01) were excluded. Normalization was performed for each microarray by using the Lumina package. A log transformation to base two was also performed on the normalized data.

In order to determine the expression of various genes of interest, quantitative polymerase chain reaction (qPCR) was performed. Briefly, cDNA was diluted and combined with SYBR green (BioRad) and corresponding primers; qPCR was then performed using BioRad’s CXF Connect Real Time machine with the following protocol: initial activation stage of 10 min at 95 °C, followed by 40 cycles of 3 min at 95 °C and 30 s at 60 °C. After these cycles were completed, temperature was brought to 65 °C for 5 min and then 95 °C for 5 min. Primers were obtained from Integrative DNA technologies (IDA; Coralville, IA, USA).

**Table:** 

**Gene**	**Direction**	**Primer sequence (5′-** **>** **3′)**
IL8 (CXCL8)	Forward	AAG CTG GCC GTG GCT CTC TTG
	Reverse	AGC CCT CTT CAA AAA CTT CTC
Olig2	Forward	GCT GCG TCT CAA GAT CAA C
	Reverse	AGT CGC TTC ATC TCC TCC A
SERPINB2	Forward	GAA GCA GGA AAG CAG AAA GAA G
	Reverse	ACT GCC ACA CAG GAA GAT ATA C
IL6R	Forward	CTC CTG CCA GTT AGC AGT CC
	Reverse	TCT TGC CAG GTG ACA CTG AG
BMP2K	Forward	TGT TGC TGC TGT GTT GCA TGA
	Reverse	TAT TGG GTC AGG GAC CTC CTC A

### Western blot analysis

In order to analyze protein expression in total extracts, cells were dissociated using trypsin after the appropriate number of days following treatment, washed with PBS, and resuspended in mammalian protein extraction reagent (M-PER; Thermo Fisher) supplemented with protease and phosphatase inhibitor (PPI; Thermo Fisher) and EDTA (Thermo Fisher). Cells were then sonicated in a water bath sonicator for 30 s, followed by a resting phase of 30 s, for a total of five cycles. Lysates were centrifuged at 21,000 × *g* for 10 min in a temperature-controlled centrifuge held at 4 °C. Supernatants were collected and protein concentration was determined by Pierce bovine serum albumin (BSA) assay (Thermo Fisher). In the case of nuclear and cytoplasmic fractionation, a cytoplasmic and nuclear protein extraction kit was used (Pierce; Thermo Fisher). Briefly, cells were dissociated and washed with PBS. Next, cells were pelleted and resuspended in ice-cold cytoplasmic extract buffer, and extraction was performed according to the manufacturer’s instructions. After collecting the cytoplasmic contents, the remaining nuclear pellets were pelleted and resuspended in ice-cold nuclear extraction buffer. Nuclear extraction was completed as instructed. All samples were stored at −80 °C when not in use.

Western blot samples were then made with equal amounts of protein in sodium dodecyl sulfate buffer (SDS sample buffer; Alfa Aesar, Wood Hill, MA, USA) supplemented with beta-mercapto-ethanol and boiled at 95 °C for 10 min. Proteins were then run through 10% SDS-polyacrylamide (SDS-PAGE; made in house) by gel electrophoresis using BioRad equipment (Hercules, CA, USA). Semidry transfer was then performed to transfer proteins onto polyvinylidene difluoride (PVDF) membranes (Millipore, Darmstadt, Germany) using a BioRad transfer machine. Membranes were washed three times in PBS for 10 min each and then blocked with Tris-buffered saline (TBS) containing 0.05% Tween20 (Sigma Aldrich, St. Louis, MO, USA) plus 5% powdered milk to limit non-specific binding. Primary antibody solutions were made using 5% BSA solutions supplemented with sodium azide. Membranes were incubated overnight on a shaker at 4 °C. The following antibodies were employed: mouse anti-IL-8 (R&D), rabbit anti-c-Myc (Cell Signaling, Danvers, MA, USA), rabbit anti-Sox2 (Cell Signaling), rabbit anti-Nanog (Cell Signaling), rabbit anti-LIN28A (Cell Signaling), rabbit anti-KLF4 (Cell Signaling), mouse anti-β-actin (Abgent, San Diego, CA, USA), rabbit anti-EZH2 (Cell Signaling), mouse anti-phosphoEZH2 (AbCam, Cambridge, UK), rabbit anti-Bmi1 (Cell Signaling), rabbit anti-ring1A (Cell Signaling), rabbit anti-SUZ12 (Cell Signaling), rabbit anti-H3 (Cell Signaling), rabbit anti-H3K4methyl3 (Cell Signaling), rabbit anti-H3K27acetyl (Cell Signaling), rabbit anti-H3K27methyl3 (Cell Signaling), rabbit anti-H3K36methyl3 (Cell Signaling), and rabbit anti-H2A-Ubiquitin (Cell Signaling). The following day, membranes were washed and then incubated in appropriate horseradish peroxidase-conjugated secondary antibodies, diluted 1:4000 in 5% milk. Membranes were then washed in TBS-T. Enhanced chemiluminescence (ECL; Clarity ECL, BioRad) was added to each membrane, and images were developed using X-ray film (General Electric, Boston, MA, USA). All densitometry analysis was performed using ImageJ (National Institutes of Health). β-Actin levels were determined for all western blots to ensure proper loading of gels.

### Coimmunoprecipitation

For coimmunoprecipitation (Co-IP) experiments, proteins were extracted and quantified as described above. Then 50–100 µg of proteins were incubated with primary antibody overnight at 4 °C with gentle rocking. The next day, anti-rabbit IgG antibodies conjugated to agarose beads were added to the cell lysates and incubated for at least 1 h at 22 °C. Next, the mixture was spun down and washed several times in PBS. Finally, proteins were eluted from the mixture and loaded into gels, as described above.

### Flow cytometry analysis

For in vitro experiments, cells were collected at serial time points after the beginning of treatment (days 2, 4, 6, and 8), and fresh surface staining was performed. Next, cells were treated with fixation and permeabilization buffers (eBioscience, San Diego, CA, USA) according to the manufacturer’s instructions. For those cells that were collected based on surface expression, no fixation or permeabilization was performed to maintain cell integrity. After this fixation, intracellular staining was performed overnight, followed by triplicate washing and the addition of appropriate secondary antibodies. In vivo studies began with the killing of tumor-bearing mice and immediate removal of the whole brain. Brains were washed in ice-cold PBS, and then bisected down the longitudinal fissure and right brains (tumor-bearing) were passed through a 70 µM strainer. These single cell suspensions were then incubated in ACK lysis buffer (Lonza, Walkersville, MA, USA) for 5 min at 20–25 °C to lysis any blood cells. After washing with PBS, cells were stained as in in vitro experiments. Human leukocyte antigen (HLA) staining was used to identify human tumor cells. All cells were collected in PBS supplemented with 1% BSA (Fisher Scientific, Fair Lawn, NJ, USA) and sodium azide and kept on ice until read.

The following antibodies were used: anti-HLA-PB (1:200; BioLegend, San Diego, CA, USA), anti-CD133-APC and anti-CD133-PE (2:100; Miltenyi Biotec, Auburn, CA, USA), anti-CD15-APC (5:100; BioLegend, San Diego, CA, USA), anti-IL-8 (R&D Systems, Minneapolis, MN, USA), anti- CXCR1 (R&D Systems, Minneapolis, MN, USA), and anti-CXCR2 (R&D Systems, Minneapolis, MN, USA). In addition, we used secondary antibodies, such as anti-rabbit IgG-FITC (1:500; Invitrogen, Waltham, MA, USA) and anti-mouse IgG-PB (1:500; Invitrogen, Waltham, MA, USA). ​Samples were run on BD LSRFortessa 6-Laser FACS analyzer (WHERE) and analyzed utilizing FlowJo software (TreeStar, Ashland, OR, USA).

For reporter cells, antibody staining was not performed. Rather, cells were dissociated using trypsin and washed with PBS. They were then resuspended in PBS with 1% BSA and sodium azide and kept on ice until analysis.

### Enzyme-linked immunosorbent assay

ELISAs were performed to determine the level of IL-8 protein in cell supernatants. After the noted number of days since treatment, supernatants were collected and centrifuged at 1200 × *g* for 5 min to pellet any cellular debris. Supernatants were then collected in fresh tubes. ELISAs were obtained from eBioscience and performed according to the manufacturer’s instructions. In summary, supernatants were collected from culture flasks on the appointed day. Supernatants were then centrifuged to pellet any floating cell or debris. Supernatants were collected and placed into clean microcentrifuge tubes. Samples were then added to ELISA plates that had been coated overnight at room temperature with capture antibody diluted in coating buffer, washed five times, and blocked for 1 h. Initial optimization runs of supernatants from non-treated cells showed that GBM43 expressed high levels of IL-8, and that supernatants needed to be diluted 1:20 to ensure signaling within the range of our standard curve; all other cell lines were diluted 1:5. After 2 h of incubation at room temperature, supernatants were removed and the ELISA plate was washed five times. Detection antibody was then added for 1 h, followed by washing, and the addition of avidin–horseradish peroxidase solution for 30 min. The plate was then washed seven times to ensure no false-positive signals were generated. Tetramethylbenzidine (TMB) solution was added and incubated for 15 min. Reactions were halted using 1 N hydrochloric acid. Plates were immediately read using a BioTek plate reader (Abs_450 nm_–Abs_570 nm_). Standard curves were generated and IL-8 concentration was determined.

### Immunohistochemistry

After whole brains were removed from animal skulls, they were washed in ice-cold PBS. Brains were then flash frozen in optimal cutting temperature compound (OCT; Electron Microscopy Sciences, Hatfield, PA, USA). Sections, 8 -microns thick, were obtained by cryostat (Leica Biosystems, Wetzler, Germany) and kept frozen at −30 °C until IHC was begun. Staining for IL-8 was performed as follows: sections were allowed to dry at 22 °C for 30 min. Excess OCT compound was then scrapped away from the margins, and an Immuno-Pen was used to create a border around each section. After one wash with ice-cold PBS, sections were fixed with 4% paraformaldehyde (Boston BioProducts, Boston, MA, USA) for 10 min. Sections were then washed three times with ice-cold PBS. A solution of 1% BSA and 0.3% Triton-X100 was then placed on top of each section for 1 h to permeabilize and block the section. Primary mouse anti-IL-8 antibodies (R&D) diluted in 1% BSA solution with 0.3% Triton-100 were then added and sections were incubated overnight at 4 °C. The following day, antibodies were removed and sections were washed three times with ice-cold PBS. Appropriate secondary goat anti-mouse IgG conjugated to FITC was diluted 1:2000 in 1% BSA with Triton-X100 and incubated for 1 h at 22 °C. Sections were then washed with ice-cold PBS three times. Fluro-Gold with DAPI (Thermo Fisher) was gently applied to each section and coverslips were carefully placed on top.

A Leica microscope was utilized for IHC analysis (Leica). For each section, tumors were identified by cell morphology and density. A blinded experimenter analyzed the slides for IL-8 expression and generated each image. ImageJ (National Institutes of Health) was used for final image processing and the generation of images for publications.

### Human sample histology

Human primary and matched recurrent GBM tissues were obtained from the Northwestern University’s Nervous System Tumor Bank. All patients were consented according to the Institutional Review Board (IRB) policies prior to the obtainment of samples. Samples were formalin-fixed and paraffin-embedded (FFPE). Immunohistochemistry of tumor samples was performed on 4-μm-thick sections heated at 60 °C for at least 1 h. Staining for IL-8 was carried out manually, and antigen retrieval was performed with a BioCare Medical Decloaking Chamber using high (LC3) or low pH antigen retrieval buffer from Dako. Primary antibodies were incubated for 1 h at room temperature. A secondary antibody was EnVision-labeled polymer-HRP (horseradish peroxidase) anti-mouse or anti-rabbit as appropriate. Staining was visualized using 3, 3′-diaminobenzidine (DAB) chromogen (Dako, K8000).

IL-8 immunohistochemical results on TMAs were semiquantified on a relative scale from 0 to 3, with 0 = negative and 3 = strongest (see Supplementary Fig. 1). Each tumor was represented by three separate cores on three separate blocks.

### Bioinformatics analysis

We utilized the publicly available The Cancer Genome Atlas (TCGA) GBM database for all examination of gene expression. We performed the following analyses: Correlation between IL-8 and all the other genes was determined by Pearson correlation coefficients. Those genes with coefficients > 0.5 or < −0.5 and false discovery rate (FDR) < 0.05 were selected to be correlated with IL-8. Then non-negative matrix factorization (NMF) was employed to identify clusters of all the genes that are correlated with IL-8 using the R package “NMF”^8^. Brunet algorithm was used to estimate the factorization. We performed 40 runs for each value of the factorization rank r in range 2:7 to build consensus map. The optimal cluster was determined by the observed cophenetic correlation between clusters, and validated by silhouette plot and principle component analysis (PCA). Function “aheatmap” was used for plotting the heatmap and clustering with “euclidean” as the distance measure and “complete” as the clustering method.

Differences of IL-8 expression among different WHO grade and subtypes were examined using one-way ANOVA, and followed by Bonferroni correction for the multiple comparison.

GBM patients were stratified into IL-8-upregulated and IL-8-downregulated groups based on IL-8 gene expression using quartile (Q1, Q3) as split points. Survival curves were generated via Kaplan–Meier method, and compared by log-rank test.

For clinical factor comparison, we used the TCGA U133a dataset. Cox proportional hazards model with stepwise variable selection was conducted to examine whether IL-8 could be independent factor for predicting survival with major clinical variables adjusted. C index (95% CI) or C statistics was provided to see how well the models are fitted, and likelihood ratio test was conducted to compare the multivariable models with and without the targeted variable.

For expression localization, we utilized the Ivy Glioblastoma Atlas Project (IVY GAP; Allen Institute for Brain Science, Seattle, WA, USA) and their online platform (glioblastoma.alleninstitue.org).

ChIP-Seq reads underwent FastQC quality analysis after sequencing and no abnormalities were detected. Alignment was done using Bowtie2 software, and peak calling was performed using the MACS2 CallPeak function with *p*-value set to 0.05. All ChIP-Seq data were visualized using Integrated Genomics Viewer (IGV).

### Extreme limiting dilution analysis and neurosphere assays

PDX cells fresh from the flank of nu/nu mice were washed with PBS and plated in serial dilutions 200, 150, 100, 50, 25, 12, 6, 3 cells per well, 12 wells per dilution, in neurobasal media (Gibco cat. no. 21103049, Thermo Fisher) supplemented with the following growth factors: B27 (without vitamin A, Invitrogen), basic fibroblast growth factor (10 ng/mL; Invitrogen), epidermal growth factor (10 ng/mL; Invitrogen), and N2 (Invitrogen) treated with either PBS or IL-8. A blinded experimenter examined each well after 7 and 14 days and counted the number of formed neurospheres with a diameter greater than 20 cells. These counts were analyzed using the Walter + Eliza Hall Institute of Medical Research outline platform (http://bioinf.wehi.edu.au/software/elda/). This platform allows for the determination of stem cell frequency, as well as quantification of differences between the IL-8 treated and non-treated samples.

### Generation of shRNA constructs

In order to knockdown the expression of IL-8, short-hairpin RNA (shRNA) constructs were obtained commercially (Genecopoeia, Rockville, MD, USA). All shRNA constructs expressed IL-8 shRNA under the control of a CMV promoter and included a GFP construction for simple identification of successfully transfected cells. Plasmids for these constructs were packaged into lentiviral vectors using X293 cells. Briefly, plasmids and all necessary transfection reagents were added to low-passage X293 cells growing as adherent cultures in DMEM media fortified with 10% FBS and 1% P/S antibody mixtures. After 3 days, supernatants were collected and ultracentrifugation was performed. Viral titration was determined by sequential dosing of collected viruses in X293 cells, followed by analysis of GFP expression. After determination of viral titer, human glioma and PDX cells were transfected with 25 IU of the lentiviral vector. This transfection was performed in suspension for 30 min at 22 °C with gentle agitation every 5 min. For in vitro experiments in human glioma cells, cells were propagated and GFP + populations were purified using FACS sorting. IL-8 knockdown was confirmed via ELISA and/or FACS analysis.

### Cell cycle analysis

These analyses were completed using the propidium iodide/RNase staining buffer (BD Pharmingen, cat. no. 550825) according to the manufacturer’s guidelines. Briefly, after the desired number of days of treatment with either DMSO or TMZ, cells were dissociated from the plate and washed with PBS. They were then treated with 70% ethanol solution as a fixative, followed by permeabilization. Cells were then treated with RNase and proteases to ensure maximum DNA staining. After incubation with these reagents, cells were washed thoroughly with PBS. Then, cells were stained with propidium iodide for 30 min at 4 °C. After washing, cells were analyzed by flow cytometry. Gating strategy was as follows: SSC-A and FSC-A were plotted and cellular debris was excluded. Then, SSC-W and FSC-W were plotted to identify only single cells. Then, SSC-A and PI stainings were plotted. Unstained controls were used to establish background signal. Finally, PI staining was plotted as a histogram. DNA content was assayed, and the progression of the cell cycle was determined based on the histogram plot. Any aberrations caused by the introduction of shRNA constructs were noted and these constructs were excluded.

### Statistical analysis

All statistical analyses were performed using the GraphPad Prism Software v4.0 (GraphPad Software, San Diego, CA, USA). Where applicable, one-way ANOVA, unpaired *t* test, and log-rank test were applied. Survival distributions were estimated with the Kaplan–Meier method. A *p*-value < 0.05 was considered statistically significant.

## Results

### Therapeutic stress increases IL-8 expression in vitro and in vivo

**Fig. 1:**
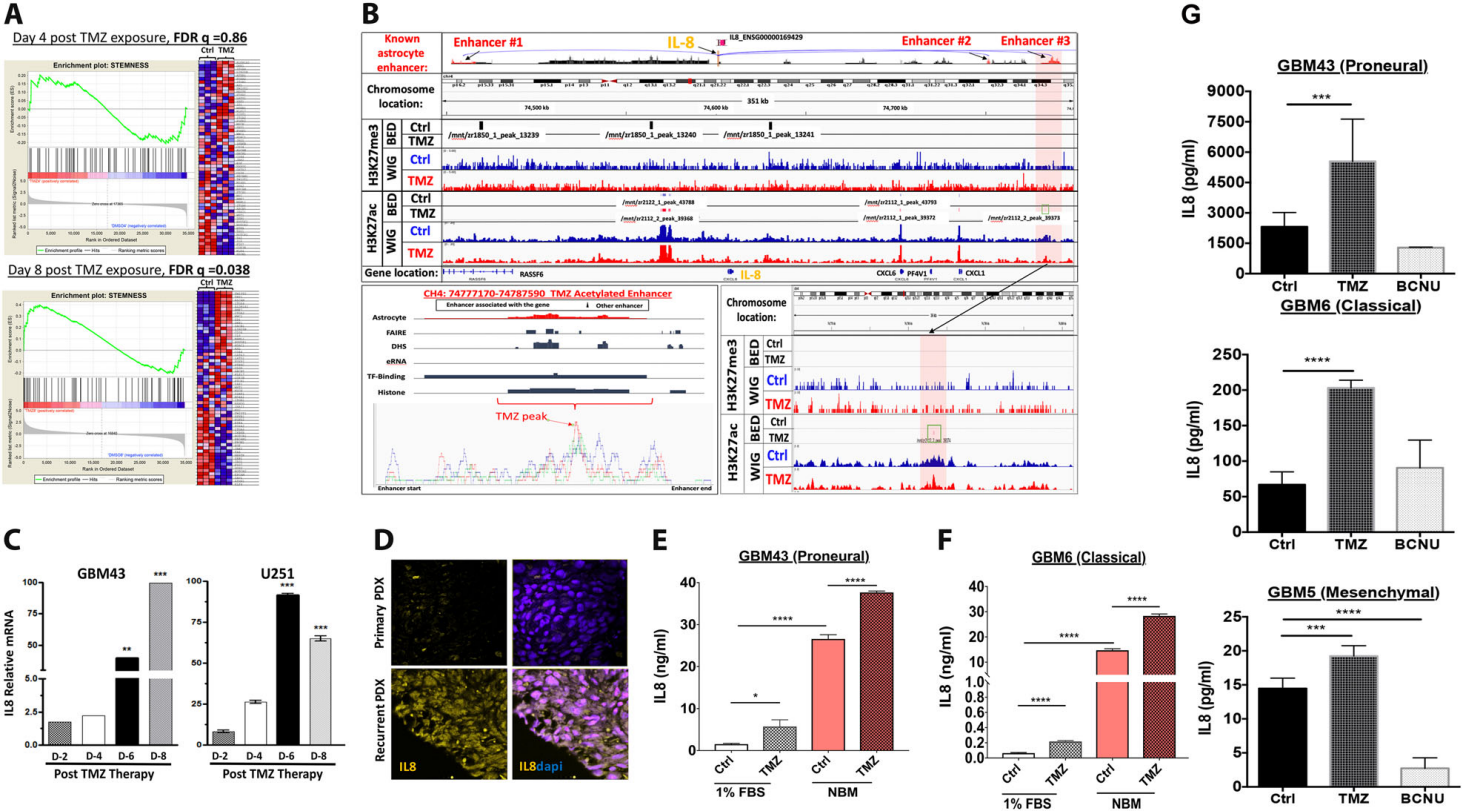
Therapeutic stress increases IL-8 expression. **a** PDX GBM43 cells were treated with TMZ (50 μM), and RNA collected after 4 or 8 days. Microarray analysis using Affymetrix platform was performed. Gene set enrichment analysis (GSEA) for genes known to support and maintain the GIC phenotype^13^ and demonstrated significance increase after 8 days [day 4 FDR q 0.82 and day 8 FDR 0.08 and FWER p-value 0.046, TMZ-treated cells as compared with DMSO controls]. **b** Whole-genome ChIP-seq analysis of H3K27 trimethylation (H3K27me) and H3k27 acetylation mark (H2K27ac) on PDX GBM43 following 4 days treatment with TMZ or vehicle control. Top two tracks represent the BET file showing significant enrichment of histone mark relative to DMSO control. Bottom two lanes represent WIG file showing significant enrichment peak. Analysis of three established astrocyte enhancer tracks for the IL-8 gene showed no changes in H3K27me3; however, they did show significant change in #3 enhancer region [Chr. 4 :74783222–74783418, fold enrichment 3.2 relative to IgG input. *p* < 0.0001. FDR = 0.004]. Right bottom inset image shows zoomed in view of the #3 enhancer region. Left bottom inset image represents overlayed peaks for TMZ and DMSO control. **c** Expression of IL-8 mRNA after exposure to 50 µM TMZ was determined by quantitative real-time polymerase chain reaction (qPCR) after treatment with TMZ across 8 days. All IL-8 values were normalized to glyceraldehyde 3-phosphate dehydrogenase (GAPDH). Bars represent means from three independent experiments and error bars represent the standard deviation. Multiple Student's *t* tests were performed. **p < 0.01, ***p < 0.001. **d** Immunohistochemistry was performed on mouse brains with intracranial xenografts of GBM43. 1.5 × 10^5^ GBM43 PDX cells were implanted to establish orthotropic xenograft tumors. Animals received 2.5 mg/kg of either DMSO or TMZ for five consecutive days, beginning 7 days after tumor implantation. Animals were killed 5 days after the cessation of treatment, and whole brains were extracted, flash frozen, then sectioned (8 µm) and analyzed by immunofluorescence. 4′-6-diamidino-2-phenylindole (DAPI) stained DNA (blue) in the nuclei and allophycocyanin-conjugated secondary (orange) antibody was used against primary antibody for IL-8. Dotted lines represent the edge of the tumor based on cell density and morphology. e–f PDX GBM xenografts were harvested and immediately plated in either mild differentiation media (DMEM containing 1% FBS) or GIC maintenance media (neurobasal supplemented with FGF and EGF). Cells were then treated with either DMSO or TMZ (50 μM). After 4 days, IL-8 levels were determined by ELISA. **g** GBM cells were treated with physiologically relevant dose of TMZ (50 μM), carmustine (BCNU, 100 μM), or equimolar DMSO. After 24 h, conditioned media was collected and IL-8 levels were quantified by ELISA. Bars represent means from three independent experiments and error bars represent the standard deviation. Multiple Student's *t* tests were performed. **p < 0.01, ***p < 0.001

To investigate if Temozolomide (TMZ) chemotherapy promotes the adoption of a GIC state via cellular plasticity, gene set enrichment analysis (GSEA) using the Affymetrix platform was performed. Data from PDX GBM43 cells 4 and 8 days post-treatment with either vehicle control (DMSO) or physiological doses of TMZ (50 μM, see Supplementary Fig. 1)^9–12^ revealed a significant (FDR q = 0.08, FWER *p*-value = 0.046) enrichment of a network of genes responsible for supporting the GIC phenotype (Fig. 1a)^13^. Interestingly, gene expression revealed that interleukin-8 (IL-8) is significantly upregulated post-TMZ therapy (Supplementary Table 1). To investigate epigenetic plasticity during TMZ therapy, we performed genome-wide ChIP-Seq analysis of TMZ-treated PDX GBM43 cells for histone 3 lysine 27 (H3K27) acetylation (ac), a marker of open chromatin, and H3K27 trimethylation (me3), a maker of closed chromatin. TMZ significantly augments H3K27ac levels, but not H3K27me3 levels, at an IL8 enhancer locus identified in astrocytes. (Fig. 1b, chromosome 4: 74783222–74783418, fold enrichment 3.02 as compared with input, *p*-value < 0.0001, FDR = 0.004). Therefore, TMZ may promote a GIC state by altering the epigenomic landscape of GBM. Quantitative polymerase chain reaction (qRT-PCR) confirmed that TMZ treatment increased IL-8 mRNA levels in a time-dependent manner (Figure 1c) (***p < 0.0001). To validate this effect in vivo, immunofluorescence analysis was performed on a previously established orthotropic recurrent GBM model, which confirmed that recurrent tumors had increased IL-8 expression (Fig. 1e). Considering the ability of therapy to induce both IL-8 expression and a GIC state, we examined if alterations in cell state can influence such expression without therapy. PDX lines were cultured in GIC maintenance media (neurobasal media supplement with appropriate growth factors) or differentiation condition media (1% FBS) with or without TMZ (50 μM). Even without any chemotherapy, culturing GBM PDX lines in the GIC maintenance media significantly elevated the expression of IL-8 expression measured by enzyme-linked immunosorbent assay (ELISA, Fig 1c) (****p < 0.0001). Proneural subtype GBM43 and classical subtype GBM6 expressed about 20- and 200-fold higher IL8, respectively, in the GIC maintenance media. TMZ exposure induced IL8 expression in both culture conditions (****p < 0.0001). Moreover, this induction was specific to TMZ, as another anti-glioma alkylating agent BCNU failed to promote IL8 expression in any GBM lines tested (***p < 0.001 and ****p < 0.0001, Fig. 1g). Based on these observations, we investigated the role of IL-8 in promoting therapy-induced cellular plasticity and disease recurrence. Critically, we utilized differentiation condition media (1% FBS or neural basal media with BMP2), as these conditions initiate differentiation of GBM cells and enable us to observe how stimuli induce dedifferentiation to the GIC state during therapy^5,7^. Culturing cells in a GIC-promoting media (neurobasal supplemented with EGF and FGF) would be inappropriate for this study, as it would force the cells to a GIC state and mask GIC-inducing effects of our experimental manipulations.

### In silico analysis establishes IL-8 importance in GBM progression and patient outcomes

**Fig. 2:**
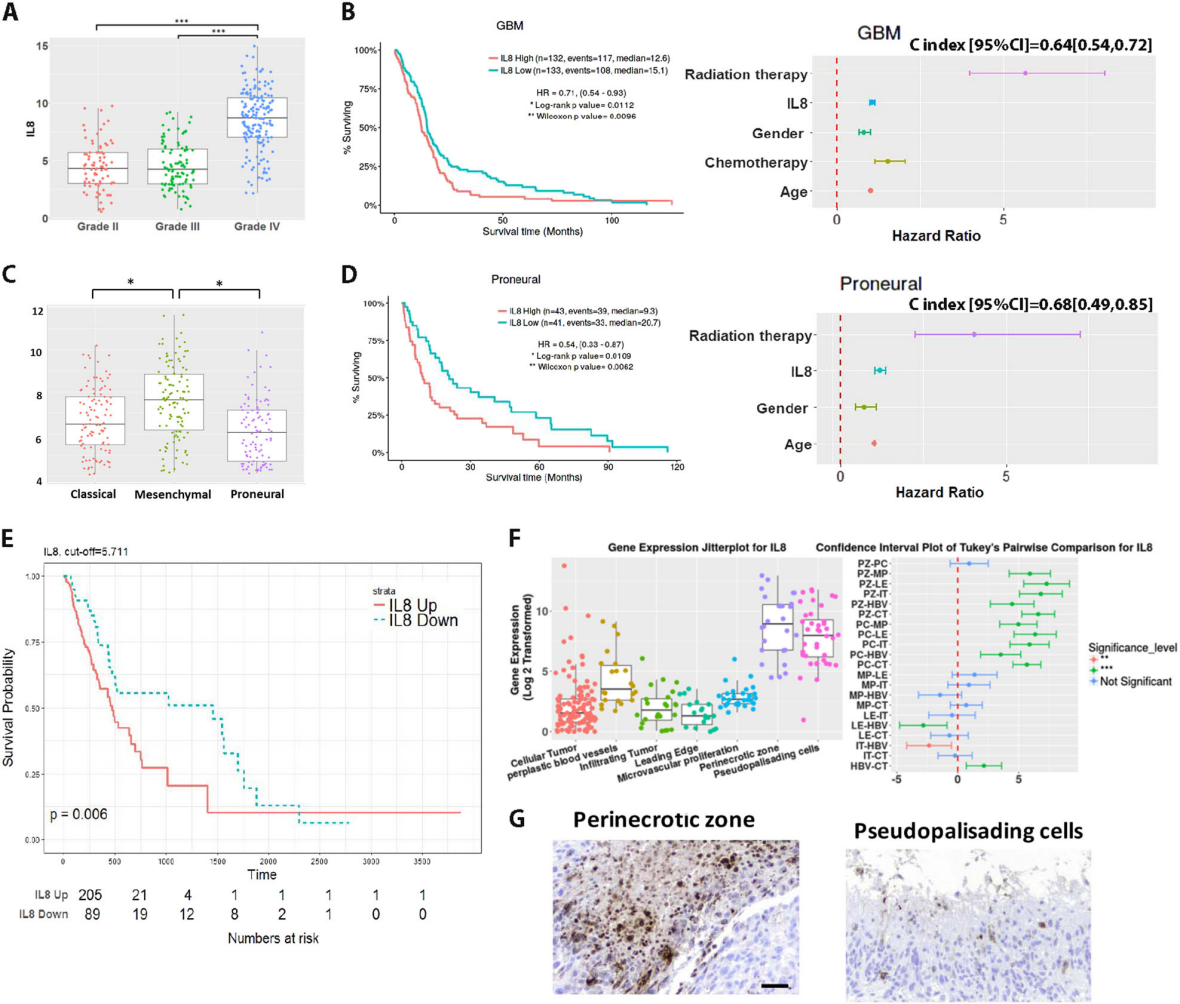
Bioinformatic analysis reveals IL-8 as a potential participant in Glioblastoma progression and outcomes. **a** *IL-8* mRNA expression levels were analyzed using the Affymetrix U133a platform on the Cancer Genome Atlas (TCGA) for different WHO grades of glioma. GBM (Grade IV glioma) patients had higher *IL-8* expression level than low-grade (Grade II, Grade III) glioma patients. **b** All GBM patients were stratified into IL-8-upregulated and IL-8-downregulated groups based on IL-8 gene expression using quartile (Q1, Q3) as split points. High expression of IL-8 correlated with reduced median survival. Survival curves were generated via the Kaplan–Meier method and compared by log-rank test. **p < 0.01. Multivariate stepwise Cox proportional hazards model with stepwise variable selection was conducted to examine whether IL-8 could be an independent factor for predicting survival with major clinical variables adjusted. This analysis confirmed that IL-8 was an independent prognostic factor for survival in all GBM patients (HR [95% CI]: all GBM 1.07 [1.01, 1.14], *p* = 0.0467). **c** Index (95% CI) or C statistics are provided. **c** Within grade IV glioma subtypes, proneural GBM patients had the lowest level of IL-8 expression. Boxplots represent means and interquartile range. One-way ANOVAs with Bonferroni correction for the multiple comparisons were performed. *p < 0.05, ***p < 0.001. **d** Patients with proneural GBM were stratified into IL-8-upregulated and IL-8-downregulated groups based on IL-8 gene expression using quartile (Q1, Q3) as split points. Kaplan–Meier survival curves and multivariate stepwise Cox proportional hazards models were generated as in B. **e** In TCGA database (U133a) GBM patients with IL-8-downregulated also have a longer time to recurrence, compared with IL-8-upregulated patients. **f** The Ivy Glioblastoma Atlas Project (IVY GAP) was employed to determine the location of IL-8 in glioblastoma samples. Each column represents the data for one biopsy from a tumor. Microdissection for the noted anatomically portions of the tumor and subsequent mRNA extraction and expression analysis demonstrated that IL-8 is upregulated in the perinecrotic zone and pseudopalisading cells. Heatmap illustrates most significantly and differential expressed genes with a false discovery rate < 0.01. mRNA expression in each anatomical compartment were compared. IL-8 was significantly upregulated in the perinecrotic zone. Bars represent means from three independent experiments and error bars represent the standard deviation. Multiple Student's *t* tests were performed. ***p < 0.001. **g** Brain tumor samples from primary biopsies or surgical resections were stained for IL-8 at the Northwestern Brain Tumor Tissue Bank. Histological and morphological analysis confirm that IL-8 is present in the perinecrotic zone and pseduopalisading cells. Scale bar 50 microns

In order to examine the contribution of IL-8 to GBM clinical progression, we employed the Cancer Genome Atlas (TCGA) patient gene expression dataset, including wild-type and IDH mutation tumors. Analysis showed that IL-8 transcript expression is elevated in World Health Organization (WHO) Grade IV glioma (GBM) (Fig. 2a, IL-8 transcript expression, Grade IV vs. Grade II *p* *<* 0.0001; Grade IV vs. Grade III, *p* *<* 0.0001). Next, GBM patients were stratified based on IL-8 mRNA expression using quartile (Q1, Q3) split points; the lowest IL-8 quartile exhibited significantly higher median survival (Fig. 2b) (All GBM: IL-8-down 15.1 months, IL-8-up 12.6 months, hazard ratio (HR) [95% CI] = 0.71 [0.54, 0.93], log-rank *p*-value = 0.0112). This association between IL-8 and survival was especially pronounced in patients with proneural GBM tumors (Fig. 2d) (proneural subtype: IL-8-down 20.7 months, IL-8-up 9.3 months, HR [95% CI] = 0.54 [0.33, 0.87], log-rank *p*-value = 0.0109). Multivariable stepwise Cox proportional hazards model confirmed IL-8 as an independent prognostic survival factor in all GBM patients, specifically those with proneural tumors (Figs 2b–d, right panel) (HR [95% CI]: all GBM 1.07 [1.01, 1.14], *p* = 0.0467; proneural subtype 1.19 [1.04, 1.37], *p* = 0.014) independent of IDH mutation status (Supplementary Figure S2). Finally, average recurrence time in GBM IL-8-downregulated patients is substantially longer than in IL-8-upregulated patients (IL-8-down 47.9 months, IL-8-up 15.5 months, hazard ratio (HR) = 0.53 [0.34, 0.84], log-rank *p-*value = 0.006) (Fig. 2e).

To investigate IL-8 expression patterns across different tumor compartments, we utilized the Ivy Glioblastoma Atlas Project (IVY GAP)^14^, which demonstrated that IL-8 mRNA is elevated in pseudopalisading cells and the perinecrotic zone, two areas linked to the GIC subpopulation (Fig. 2f). All of these data further justify our interest in IL-8 as a critical participant in GBM progression and therapy-induced plasticity.

### Immunohistochemical analysis to IL-8 expression in matched primary recurrent GBM tissue

**Fig. 3:**
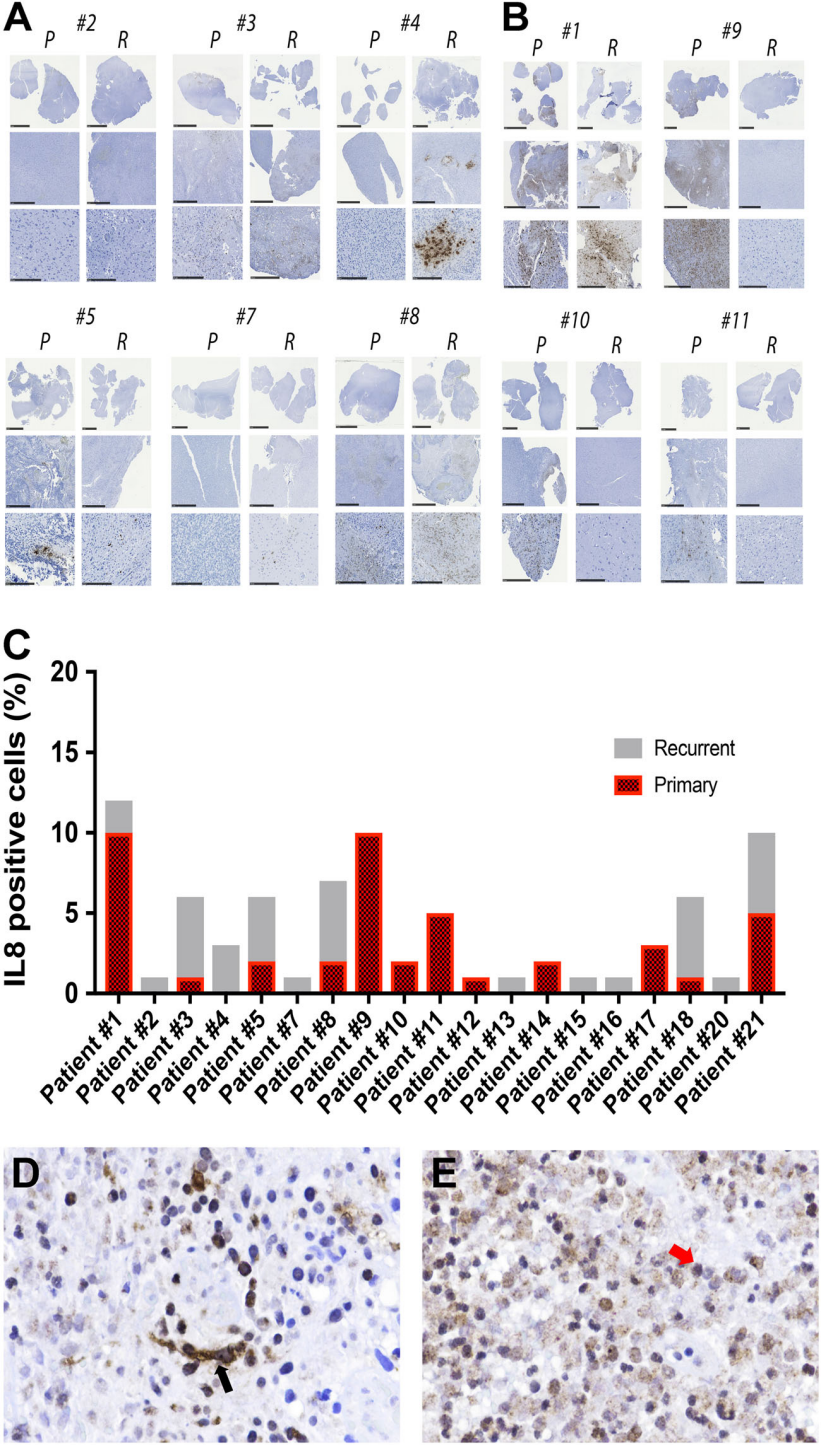
Immunohistochemical analysis of IL-8 expression in the matched primary and recurrent GBM patient samples. **a** Representative immunohistochemical staining for IL-8 in the matched primary (P) and recurrent (R) clinical GBM samples. Top row low-powered and bottom row high-powered magnification of IL-8 staining. Scale bar for all top row images 1 mm, all middle row images 5 mm, and for the bottom row images 250 µm. These sets of patient samples show IL-8 upregulation in the matched recurrent tissues. **b** Same as previous, but this set of patient samples shows a decrease in IL-8 staining for recurrent GBM as compared with their matched primary GBM. **c** Quantitative analysis of the percent of IL-8 positive cells in the matched primary and recurrent GBM tissue. Representative immunohistochemical analysis of IL-8 expressing (**d**) tumor cells (left, black arrow) and (**e**) infiltrative macrophage (right, red arrow)

To investigate IL-8 expression in GBM tissue from patients, 75 GBM specimens from Northwestern University’s Brain Tumor Bank were subjected to immunohistochemical characterization. Pathological analyses found 65% (49/75) of GBM samples were IL-8-positive. Further, 17 matched primary and recurrent GBM tumor pairs were examined for IL-8 expression. We found elevated IL-8 in 65% (11/17) of recurrent tumors (Figs 3a–c). Critically, IHC analysis revealed high IL-8 expression in the typically hypoxic perinecrotic zone, an anatomical compartment where GIC’s are maintained 11–14 (Fig. 2f). Furthermore, both tumor cells and infiltrating macrophages express IL8 (Fig. 3d, e).

### IL-8 receptor CXCR2^+^ GBM cells acquire CD133 expression during anti-glioma chemotherapy

**Fig. 4:**
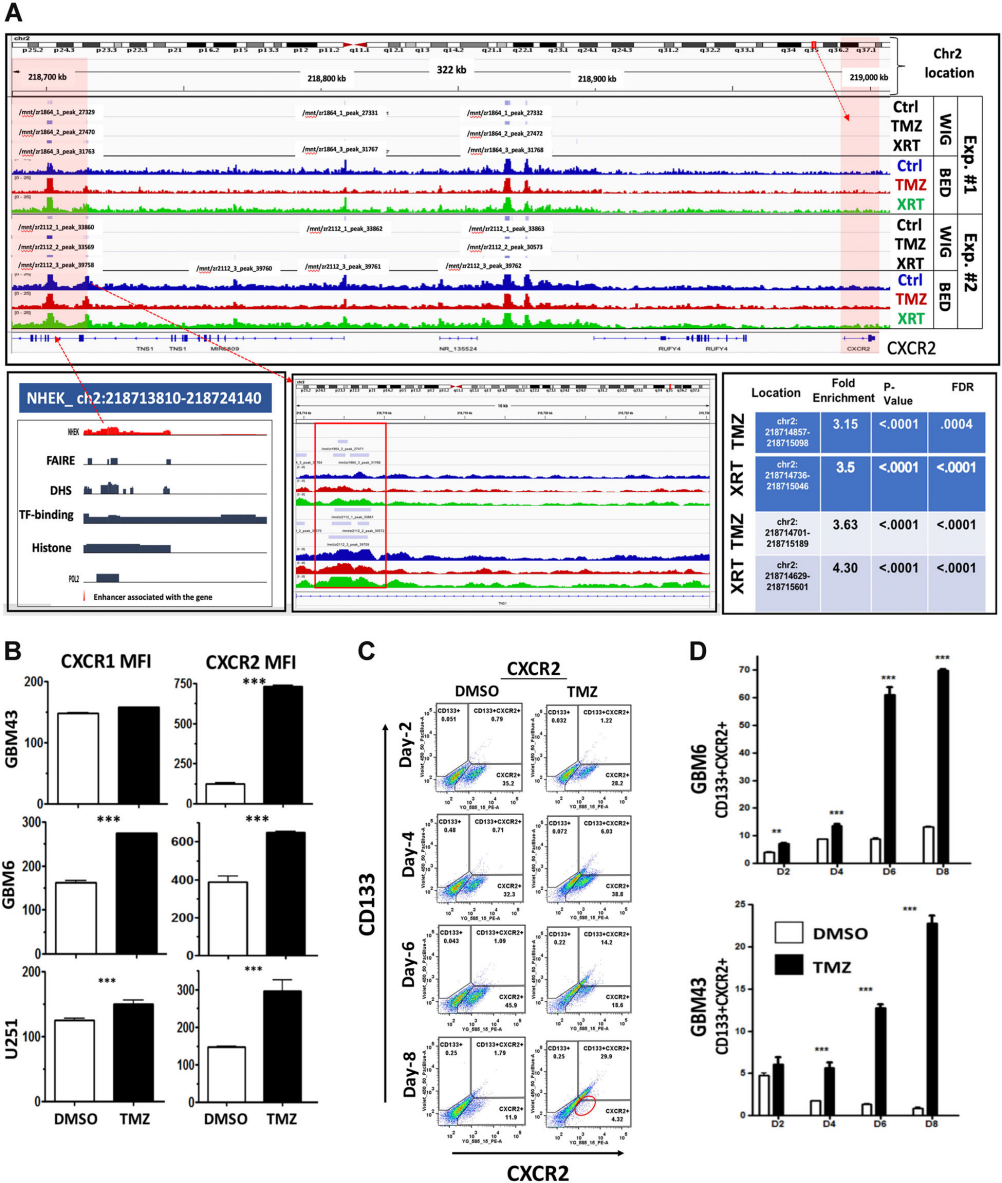
Therapeutic stress alters epigenetic status and increases expression of CXCR2, one of the major receptors for IL-8. **a** ChIP-seq analysis was performed for H3K27ac, a marker of open chromatin associated with activation of gene expression, and H3K27me, associated with closed chromatin and repressed gene expression, on PDX GBM43 cells. Cells were treated with either TMZ (50 μM) or equimolar DMSO for 4 days prior to analysis. Top track shows the location of the CXCR2 gene, with higher magnification analysis of the area shown below. TMZ-treatment led to increased H3K27ac enrichment, including in a well-established enhancer region for CXCR2 (green box and red box) [Chr2:218714857–218715098, fold enrichment 3.14 relative to IgG input, *p-*value < 0.0001, FDR 0.0004; Chr2:218714701–218715149, fold enrichment 3.63 comp. input, *p* < 0.0001, FDR < 0.0001]. Furthermore, gene body H3K27me3 was significantly reduced following TMZ [*p* = 00120 in TMZ relative to DMSO control levels]. **b** FACS analyses were performed to determine how TMZ treatment alters the levels of CXCR2 in three GBM cell lines—GBM43, GBM6, and U251. Samples were analyzed 8 days after initial treatment with either DMSO or TMZ (50 µM). All data are expressed as the mean fluorescent intensity (MFI). Bars represent means from three independent experiments and error bars represent the standard deviation. Multiple Student's *t* tests were performed. ***p < 0.001. **c** Representative FACS scattered plot analyses from GBM43 cells treated with either DMSO or 50 µM TMZ across 8 days. Circle highlights the clear shift of the CXCR2 expressing population into the CD133 + compartment. **d** TMZ treatment significantly increased expression of CXCR2 in both GBM43 and GBM6, with CXCR2 expressing cells beginning to co-express CD133 GIC markers in a time-dependent manner. All data are expressed as the percentage of total live cells. Bars represent means from three independent experiments and error bars represent the standard deviation. Multiple Student's *t* tests were performed. **p < 0.01; ***p < 0.001

Next, we set to investigate how IL-8 signaling influences GBM proliferation and cellular signaling. CXC motif chemokine receptors 1 and 2 (CXCR1 and CXCR2) are the major receptors for IL-8^15^. To investigate their role in IL-8-mediated signaling in GBM, we interrogated TCGA data. We observed that expression of both these receptors was significantly elevated in GBM tumors compared with low-grade gliomas (Figure S2-A). Analysis of our ChIP-seq data showed post-therapy accumulation of open chromatin mark at a known enhancer site^16^ for CXCR2 (Fig. 4a, *p*-value < 0.0001), as well as significant decreases in H3K27me3 levels in the gene body (Figure S2 C–H, *p*-value < 0.001). In contrast, gene body H3K27me3 levels were significantly increased at the CXCR1 gene locus (Figure S2-B). FACS analysis for CXCR1/2 demonstrated that all cell lines increased expression of both receptors post-TMZ treatment. (Fig. 4b, *p* *=* 0.00015). CXCR2 expression was also elevated in the CD133^+^ GIC population (Fig. 4c). Time course FACS following TMZ treatment revealed that a CXCR2^+^ cell population exists prior to TMZ treatment; this population rapidly gains CD133 expression during treatment (Fig. 4d). CXCR1 expression was not altered during therapy (Fig. 4b and Figure S2-I). Analysis of the downstream effectors of the IL-8/CXCR signaling cascade in PDX GBM demonstrated that IL-8 induces dose-dependent activation of canonical ERK1/2 and AKT signaling (Figure S2-J).

### IL-8 increases the self-renewing capacity of GBM cells and the expression of GIC markers

**Fig. 5:**
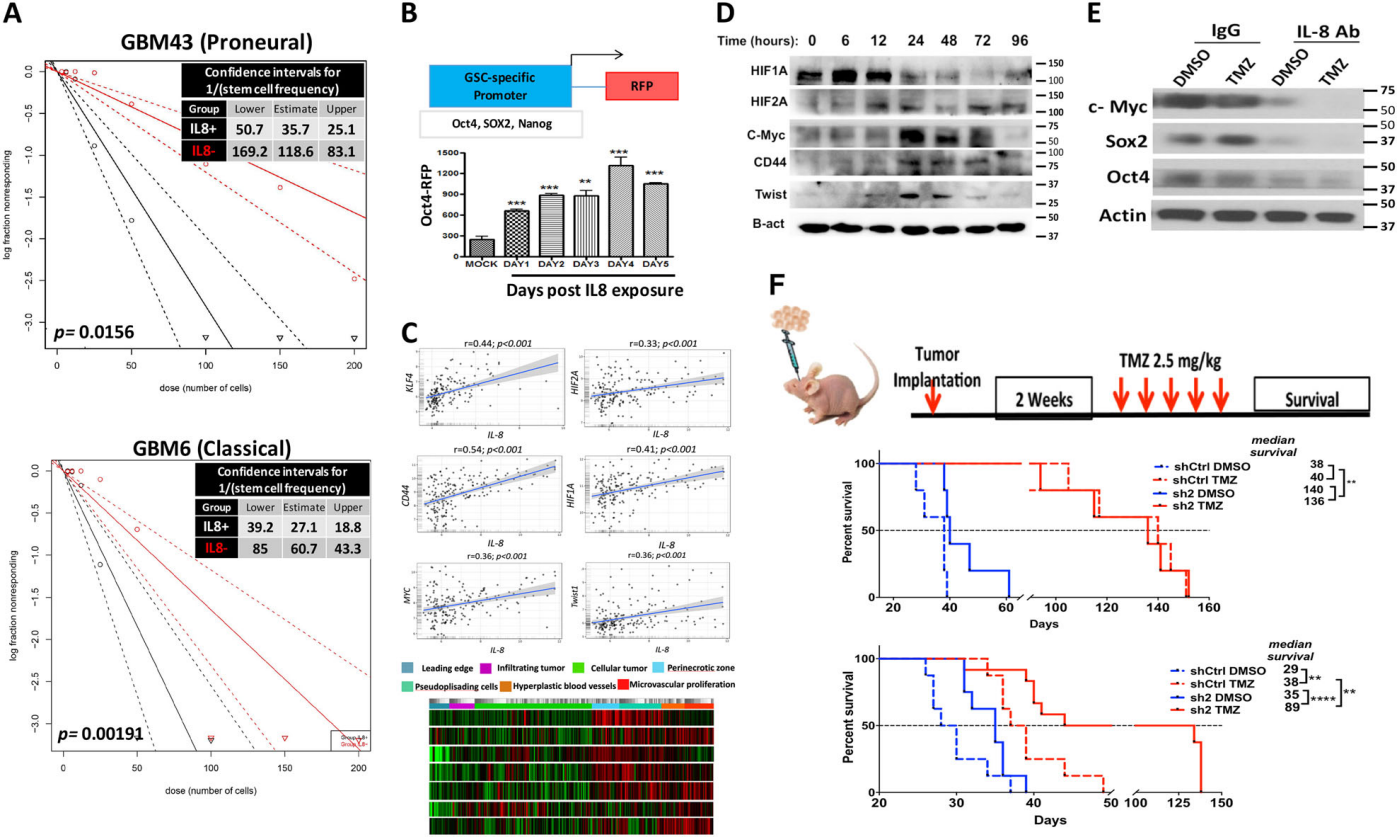
IL-8 contributes to glioma-initiating cell phenotype and contributes to GBM growth in vivo. **a** Limiting dilution neurosphere assays were performed on two cell lines—GBM43 and GBM6—after treatment with 50 ng/ml of IL-8. Stem cell frequency for GBM43 with IL-8 35.7, lower limit 50.7 and upper limit 25.1 as compared with no IL-8 118.6, lower limit 169.2 and upper limit 83.1, *p* = 0.0156; for GBM6 with IL-8 27.1, lower limit 39.2 and upper limit 18.8 as compared with no IL-8 60.7, lower limit 85 and upper limit 18.8, *p* *=* 0.001. **b** To determine the ability of IL-8 to influence cellular plasticity, we employed a reporter cell line in which RFP expression is controlled the OCT4 promoter. Cells were treated with 50 ng/ml of IL-8, and RFP expression was monitored by FACS over 6 days. Treatment increased both Oct4 and Sox2. Bars represent means from three independent experiments and error bars represent the standard deviation. Multiple Student's *t* tests were performed. **p < 0.01, ***p < 0.001. **c** The network of GIC-promoting genes in patient tumors, we selected the top GIC-associated genes activated during TMZ therapy (Fig. 1a and Supplementary Table 2) and correlated with their levels with IL-8 mRNA using the GlioVis data portal for visualization and analysis of brain tumor expression database (gliovis.bioinfo.cnio.es; dataset LeeY) 16–18. IL-8 expression was significantly correlated with critical GIC-associated genes including KLF4, CD44, HIF1A, HIF2A, Myc, and Twist expression (Fig. 5c). The IL-8 expression in different anatomical location and potential colocalization of these GIC-specific genes with areas of high IL-8 transcript level (Fig. 5c, heatmap). **d** Immunoblot analysis of endogenous glioma-initiating cell-associated transcription factors expression upon stimulation with escalation dose of IL-8 (0–100 ng/ml) for 24 h. Protein extracts of IL-8-treated PDX lines GBM43 and GBM6 were immunoblotted with antibody against several GIC markers, including c-myc, Sox2, Nanog, KLF4, OCT4, or an antibody against β-actin as a control for equal loading. **e** GBM43 PDX cells were treated with neutralizing antibody against IL-8 or control IgG antibody (100 ng/ml) prior to treatment with DMSO or 50 µM TMZ. Neutralizing antibody was added every day for 8 days,and protein extracts from this experiment were immunoblotted with antibody against c-myc, Sox2, OCT4, or an antibody against β-actin as a control for equal loading. **f** Schematic diagram of experiment design for in vivo testing. Top graph, U251 cells were infect with lentivirus (Sigma Mission shRNA) shRNA against IL-8 or scrambled shRNA (control) with 10 infectious unit/cell. In total, 2 × 10^5^ transduced cells were stereotactically injected into the right hemisphere of the brain of athymic nude mice (*n* = 8 per group, four males and four females). Two weeks after implantation, two groups of mice, control, and knock down, were treated with vehicle treated (DMSO, top curve) or TMZ (2.5 mg/kg) intraperitoneally. Survival curves were obtained by the Kaplan–Meier method, and overall survival time was compared between groups using log-rank test. All statistical tests were two-sided. Bottom graph, to examine the role of IL-8 in GBM progression in a more clinically relevant manner, next the same method was used to knockdown the IL-8 expression in GBM43 PDX line. In total, 1.5 × 10^5^ cells were injected stereotactically into the right hemisphere of the brain of athymic nude mice (*n* = 8 per group, four males and four females). Survival curves were obtained by the Kaplan–Meier method, and overall survival time was compared between groups using log-rank test

To determine if the IL-8-CXCR signaling axis promotes induction of the GIC state in GBM, we performed extreme limiting dilution assay (ELDA) on GBM6 and GBM43 cells in neurosphere media containing IL-8 (50 ng/ml). IL-8 increased GIC frequency about 3.3-fold for GBM43 and 2.3-fold for GBM6 (Fig. 5a, *p* = 0.001). We next examined how IL-8 alters the expression of known GIC-promoting genes, using our proprietary GIC-specific reporter cell line^7^. IL-8 activation significantly increased reporter activity (Fig. 5b and Figure S3-A for SOX2-RFP reporter and Nanog-RFP reporter *p* = 0.0005). To investigate how the IL-8-CXCR signaling cascade interacts with GIC-promoting genes in patient tumors, we selected the top GIC-associated genes activated during TMZ therapy (Fig. 1a and Supplementary Table 2) and correlated with their levels with IL-8 mRNA using the GlioVis data portal 16–18. IL-8 expression was significantly correlated with critical GIC-associated genes, including KLF4, CD44, HIF1A, HIF2A, Myc, and Twist expression (Fig. 5c). Ivy Gap was used to explore colocalization of GIC-specific genes to IL8 expression within tumor compartments. We observed that GIC-specific genes were correlated with IL-8 expression in both the perinecrotic zone and pseudopalisading cells (Fig. 5c, heatmap; Ivy Glioblastoma Atlas Project.). Immunoblot analysis of PDX lines exposed to IL-8 shows time-dependent induction these genes (Fig. 5d) as well as various critical GIC-associated transcription factors, such as C-myc, Nanog, Sox2, and OCT4 (Figure S3-D). Finally, to examine the role of post-therapy IL-8 in inducing the GIC-specific gene expression, we combined DMSO or 50 µM TMZ with either control IgG or anti-IL-8 neutralizing antibody. Blocking of IL-8 both reduced basal expression of GIC markers and prevented TMZ-induced increases in SOX2 and C-Myc (Fig. 5e).

### IL-8 enhances GBM growth and therapy resistance in vivo

To elucidate IL-8’s role during in vivo GBM growth, U251 cells with stable IL-8 knockdown were established using shRNA technology. IL-8 secretion was effectively knocked down by two shRNA constructs (Figure S4-A and B, *p* < 0.0005). Proliferation capacity was altered significantly in cells with the highest IL-8 knockdown compared with control population (Figure S4-C, *p* = 0.018), but cell cycle profiles remained stable (Figure S4-D and E). Athymic, immunodeficient mice then had GBM cells expressing either sh-Control or anti-IL-8 shRNA#1 implanted into the right cerebral hemisphere. Each group was then divided into two groups that received either DMSO or TMZ (2.5 mg/kg, i.p.) (*n* = 7/group). IL-8 knockdown significantly increased the median survival of animals with orthotropic GBM regardless of chemotherapy exposure (Fig. 5f, first graph median survival sh-control 38 days vs. sh#1 IL-8 140 days; hazard ratio of survival = 4.737, 95% CI = 4.190 to 101.3, *p* = 0.0021). In a clinically relevant model, PDX GBM43 (which express high basal levels of IL-8, Fig. 1c and e) were infected with a lentivirus carrying shRNA against IL-8, reducing IL-8 expression by 50% (Figure S6-A, *p* > 0.0005) after transient transfection. Implantation of these IL-8 knockdown GBM43 prolonged median survival about 38% compared with control shRNA (Fig. 5f, 2nd graph median survival for sh-control 29 days vs. sh#2 IL-8 40 days, *p* = 0.0003). Moreover, IL-8 knockdown significantly enhanced the therapeutic efficacy of TMZ and improved survival about 51 days (Fig. 5f).

### IL-8 signaling promotes epigenetic alterations in GBM

**Fig. 6:**
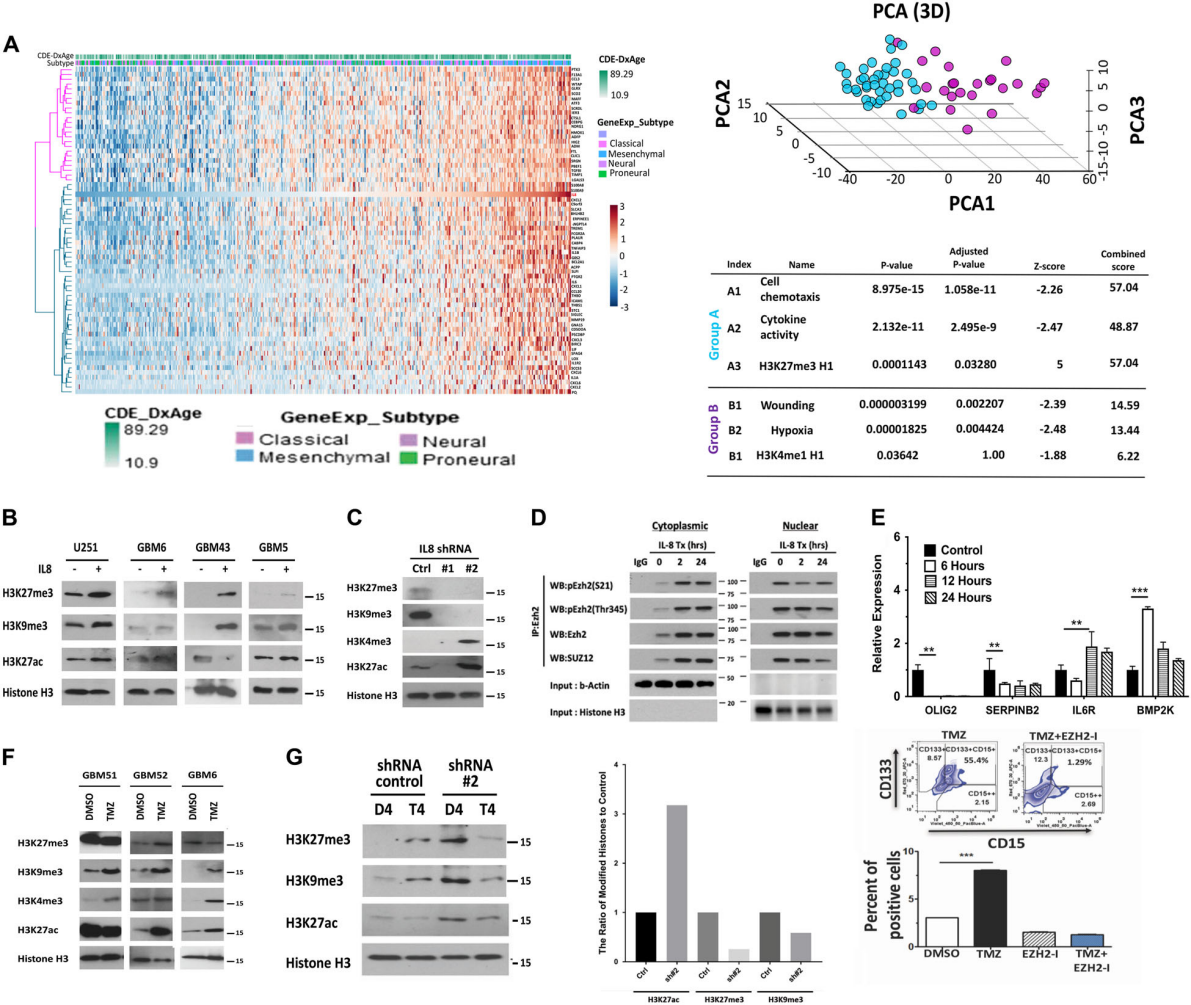
IL-8 signaling alters histone marks, promoting post-therapy epigenetic plasticity. **a** Correlation between IL-8 and 12042 genes from TCGA was determined by Pearson correlation coefficients. Sixty-eight genes with coefficients > 0.5 or < −0.5 and false discovery rate (FDR) < 0.05 were selected. Unsupervised hierarchical clustering of those genes found two clusters with the highest cophenetic coefficient at 0.95 and an average silhouette width at 0.46. Principal component analysis was used to validate these clusters. Enrichment analysis found one cluster of genes was enriched at cell chemotaxis (GO: 0060326, adjusted *p*-value 1.058e-11) and cytokine activity (GO: 0005125, adjusted *p*-value 2.495e-9), while another cluster of genes enriched at wounding (GO: 009611, adjusted *p*-value 0.002) and hypoxia (GO: 0001666, adjusted *p*-value 0.004). **b** Representative immunoblot of different histone marks. A panel of PDX lines from a different subtype of GBMs was exposed to IL-8 (50 ng/ml) for 24 h. Nuclei were extracted from the harvested cells and subjected to immunoblot analysis for suppressive histone marks H3K27 and H3K9 trimethylation (me3) and activating mark H3K27 acetylation(ac). Immunoblotting for total histone three was performed to confirm the equal loading. **c** The extracted nuclei from the U251 IL-8 knockdown cells as described in Fig. 5f were subjected to immunoblot analysis for various histone marks as described above. **d** GBM43 cells were treated with IL-8 (50 ng/ml) for 2 and 24 h, cytoplasmic and nuclear extract was prepared, cells were harvested, and immunoprecipitation assays were performed with the anti-EZH2 antibody. Immunoprecipitated protein was subjected to immunoblot analysis with antibodies against phosphor-EZH2 (S21, and Thr345), and SUZ12. β-Actin and histone 3 were used as loading controls. **e** IL-8 (50 ng/ml)-treated GBM43 PDX lines were harvested at 6, 12, and 24 h post IL-8 exposure. mRNA was extracted and subjected to reverse-transcription polymerase chain reaction (RT-PCR) analysis of *OLIG2, SERPINB2, IL6R*, *and BMP2K* transcripts. Bars represent means from two experiments in triplicate and error bars represent the standard deviation. Multiple Student's *t* tests were performed. **p < 0.01, ****p < 0.0001. **f** A panel of GBM PDX lines was treated with TMZ (50 µM) for 48 h. Nuclei were extracted from the harvested cells and subjected to immunoblot analysis for suppressive histone marks H3K27me3 and H3K9me2 and activating mark H3K27ac and H3K4me3. Immunoblotting for total histone three was performed to confirm equal loading. **g** The U251 IL-8 control and knockdown cells as described in Fig. 5a were treated with TMZ(50 µM) for 4 days. Nuclei were extracted from the harvested cells and subjected to immunoblot analysis for suppressive histone marks H3K27me3 and H3K9me2 and activating mark H3K27ac. Left, representative densitometry analysis is expressed as percent of control shRNA (shCtrl). **h** The GBM43 PDX line was treated with TMZ(50 µM) in the presence of 3-deazaneplanocin A (DZNep, EZH2-I, 5 µmol/L), a histone methyltransferase EZH2 inhibitor for 8 days. Cells were harvested and the GIC population was analyzed by FACS analysis of the CD133 and CD15 positive cells. Bars represent means from two experiments in triplicate and error bars represent the standard deviation. Multiple Student's *t* tests were performed. ***p < 0.001

To identify potential mechanisms by which IL-8 influences growth and promotes therapeutic resistance, we analyzed correlations between IL-8 and 12042 other genes using TCGA GBM patient data via Pearson correlation coefficients. Our analysis returned 68 genes with coefficients > 0.5 or < −0.5 and FDR < 0.05 that correlate with IL-8; we then conducted unsupervised hierarchical clustering. Observed cophenetic correlation determined optimal clusters, which we validated by silhouette plots and principal component analysis (PCA). Two clusters with the highest cophenetic coefficient at 0.95 and average silhouette width at 0.46 (Figure S5 and Fig. 6a) separated well upon visualization of PCA. Using the Enrichr platform, we determined that the first of these clusters (Group A) is involved in regulating cell chemotaxis (GO: 0060326, adjusted *p*-value 1.058e-11) and cytokine activity (GO: 0005125, adjusted *p*-value 2.495e-9), well-established canonical roles for IL-8^17,18^. Cluster B includes genes enriched for wounding (GO: 009611, adjusted *p*-value 0.002) and hypoxia (GO: 0001666, adjusted *p*-value 0.004), also known IL-8 connections^19,20^. Interestingly, IL-8 signaling also positively correlated with genes known to regulate epigenetic processes, specifically histone 3 lysine 27 trimethylation (H3K27me3) (GO: 0001666, adjusted *p*-value 0.03).

Trimethylation of H3K27 suppresses gene expression via recruitment of the polycomb repressor complex (PRC), predominately regulated by two methyltransferases, EZH2 and G9a^21,22^. Confirming our in silico result, treatment with IL-8 significantly increased trimethylation of H3K27, as well as another PRC complex target, H3K9^23^, in three PDX lines (Fig. 6b). Moreover, reduction of IL-8 expression via shRNA abolished methylation of the H3K27 and H3K9 residues (Fig. 6c), with dose-dependent effects (Supplementary Figure S6-B).

To further elucidate the connection between IL-8 and PRC, we examined the status of PRC members following IL-8 exposure. Phosphorylation of EZH2 in response to various extracellular stimuli remodels the epigenomic landscape, allowing cellular adaptation^24^. Specifically, phosphorylation at Thr345 enhances recognition of target genes leading to recruitment of PRC2 and suppression of transcription via H3K27me3.^25^ Contrastingly, extracellular AKT signaling suppresses the methyltransferase activity of EZH2 by phosphorylating Ser21^26^. Our previous results illustrated that IL-8–CXCR interaction could activate various downstream signaling cascades, including PI3K–AKT (Figure S6-C and D)^27^. We, therefore, examined the alteration of the phosphorylation status of EZH2 by IL-8 via immunoprecipitation (IP) in the nuclear and cytoplasmic fraction (Fig. 6d). IL-8 stimulation enhanced phosphorylation of EZH2 at both Ser21 and Thr345, exclusively in the cytoplasmic fraction. However, within 2 h of IL-8 stimulation, S21-phosphorylated (inhibited) EZH2 levels were decreased in the nucleus, while Thr345-phosphorylated (activated) EZH2 accumulated. Binding of EZH2 to SUZ12, an essential PRC protein, increased only in the cytoplasmic compartment after IL-8 exposure, while the nuclear accumulation of EZH2–SUZ12 complex gradually decreased. We conclude that within 2 h of IL-8 exposure, the PRC2 activity may increase, but by 24 h nuclear accumulation of PRC2 complex is reduced by heightened phosphorylation of EZH2 at Ser21.

To determine the functional effect of IL-8 on EZH2 activity, we analyzed genes positively regulated by EZH2 (OLIG2, SERPINB2) and negatively regulated genes IL6R and BMP2K via qRT-PCR (Fig. 6e)^28^. Remarkably, IL-8 exposure reversed expression of all four EZH2 target genes, indicating that IL-8-induced EZH2 modifications does alter gene transcription.

Next, we expanded our investigation into TMZ-induced therapeutic stress-induced alterations in histone markers that are targets of EZH2/PRC2. Treatment with TMZ induced subtype- and time-dependent global changes in epigenetic markers H3K27me3 and H3K9me3 and open-chromatin marker H3K27 acetylation (ac) (Fig. 6f). Additionally, PRC2 target H3K9me3 was upregulated in GBM6. H3K27 trimethylation and acetylation were upregulated within 48 h post-TMZ exposure and stayed elevated. H3K4 trimethylation also increased within 96 h, indicating acquisition of bivalency^29^.

Given that TZM induces IL-8 signaling and EZH2-dependent changes in histone status, we next examined the relationship between TMZ, IL-8, and EZH2 target histones. We treated IL-8-knockdown cells with TMZ for 96 h and analyzed histone status. Reduced IL-8 levels abolished methylation of EZH2 targets H3K27 and H3K9; however, H3K27ac decrease was minimal. Consequently, we suspect that chemotherapy-induced IL-8 signaling participates in EZH2-dependent epigenetic modifications during therapeutic stress. Finally, to investigate the role of EZH2/PRC2 complex activity in promoting therapy-induced cellular plasticity, the GBM43 PDX line was treated with TMZ in the presence of 3-deazaneplanocin A (DZNep, EZH2-I), a histone methyltransferase EZH2 inhibitor. DZNep abolishes the induction of a GIC population after therapy, as measured by FACS analysis of the CD133 + and CD15 + populations (Fig. 6h, *p* > 0.0005).

## Discussion

The ability of GBM cells to adapt to current therapies and generate treatment-resistant recurrences represents a critical challenge facing brain tumor researchers and clinicians. Here, we provide data that illustrates new mechanisms that may underlie this powerful ability to react to and overcome standard of care therapies. This study highlights the IL-8/CXCR2 signaling pathway as a critical player in this process and a potential target for blocking GBM cellular plasticity during therapy. Specifically, our data show: (1) therapeutic stress alters the epigenetic regulation of IL-8 leading to increased expression and secretion of IL-8; (2) bioinformatics analysis and IHC analysis of matched primary and recurrent patient tissues suggest that IL-8 significantly influences patient progression and time to recurrence; (3) therapeutic stress-induced IL-8 alters the phenotype of GBM cells, shifting them to a more GIC-like state; (4) IL-8 supports GBM aggression and resistance to chemotherapy in vivo; and (5) IL-8 signaling may influence the acquisition of GIC state via modulation of the histone modifying PRC2 complex.

Induction of cellular plasticity, is a well-established player in the formation of GBM recurrence. Indeed, we and several other groups have demonstrated how standard therapies can initiate this process and enrich GBM tumors with GIC cells^4,5,7,30^. However, targetable players in this process have yet to be identified. Here, we provide evidence that IL-8 represents one such target. Our results show that IL-8 is sufficient to induce the GIC state on its own and that it is both adequate and necessary for the adoption of GIC state during therapeutic stress.

These results highlight IL-8 signaling as a potent regulator of GBM phenotype. Not only was IL-8 able to induce the expression of CD133 and CD15, two GIC phenotypic markers, it also caused increased expression of many transcription factors known to promote the GIC phenotype, including HIF, c-myc, Sox-2, and CD44. In light of the ongoing debate regarding the precise gene expression profile of GICs, this robust induction, combined with our matched patient data, provides strong evidence that IL-8 is capable of activating cell reprogramming toward a more GIC-like state during chemotherapy.

Another key aspect of this study concerns the tumor microenvironment and how therapy can influence its composition. We show that GBM cells manipulate their microenvironment following treatment with chemotherapy. This fact further corroborates a growing body of evidence that tumor cells cultivate a pro-growth microenvironment. An interesting facet of our data is the fact that culturing GBM cells in pro-GIC conditions alone led to an increase in IL-8 levels, suggesting that tumor cells may utilize positive-feedback loops to respond to their surroundings rapidly, a potential new therapeutic target.

One open question from this study is potential non-tumor sources of IL-8 in the tumor microenvironment. While our results illustrate a robust role for GBM autocrine IL-8, it remains an open question how IL-8 from surrounding non-tumor cells might participate in the recurrence process. Cytokines from the stroma and infiltrating immune cells have been identified as regulators of tumor behavior in other cancer types, including breast cancer^31,32^. Previous work has shown that IL-8 signaling in the perivascular niche is critical to GIC behavior and phenotype^33^. In fact, our IHC analysis shows that infiltrating macrophage express IL-8 in the tumor microenvironment. Moreover, astrocytes and brain endothelial cells are both known to release cytokines, especially in response to damage^34–36^. Indeed, one of the top hits from our bioinformatics analysis of pathways correlated with IL-8 expression in patient samples was wound healing. These data and the potential involvement of non-tumor cells provide further evidence for the theory that cancer represents an “un-healing” wound and is caused by inappropriate activation of damage response and inflammatory pathways. New research will shed light on this hypothesis. In light of the fact that GBM is highly sensitive to changes in the microenvironment^2,37^ and factors secreted by nearby neurons^38^, it is highly possible that non-tumor IL-8 may participate in the processes described here.

Another open question is the specific mechanisms activating the production of IL8 during therapy. We have previously reported that therapeutic stress activates hypoxia inducible factor (HIF) signaling in GBM and promotes cellular plasticity. Evidence exists that HIF signaling can promote the synthesis and secretion of cytokines, including IL8^39–41^. The fact that IL-8 is strongly correlated with HIF levels in patient samples supports the idea that HIF and IL8 may also be linked in GBM’s response to therapeutic stress. Another potential mechanism driving IL8 induction is IKK-regulated transcription, a key process shown to regulate responses to chemotherapy in other cells^42^. Further research will illuminate the driving force of chemotherapy-activated IL8 induction and secretion.

Our data indicate for the first time that IL-8 is capable of causing alterations in the epigenetic status of key gene regulation factors, such as H3K27. While evidence increasingly shows the importance of epigenetic regulation in GBM growth and therapy resistance, the mechanisms activating these processes remain incompletely understood. IL-8 appears to act as one tumor-derived trigger for activating epigenetic responses to tumor therapy via modulation of the canonical PRC2 complex.

In sum, our data show that IL-8 is a key microenvironmental factor involved in promoting cellular plasticity in GBM. Through analysis of murine models and patient data, we illustrate the high degree of influence IL-8 holds over tumor progression. Furthermore, our work connects IL-8 signaling to the increasingly important area of epigenetic regulation of gene expression to allow tumor growth. These results highlight IL-8/CXCR signaling as a key target for GBM drug development, especially in combination with standard of care therapies.

## Supplementary information

Supplementary Figures
