# Supplementary material for: Interleukin-8/CXCR2 signaling regulates therapy-induced plasticity and enhances tumorigenicity in glioblastoma
Source: Cell Death Dis. 2019 Mar 29;10(4):292. doi: 10.1038/s41419-019-1387-6 (PMC6441047; doi:10.1038/s41419-019-1387-6)
Supplement: Supplementary file 1 — Supplementary Figures [file 41419_2019_1387_MOESM1_ESM.docx]

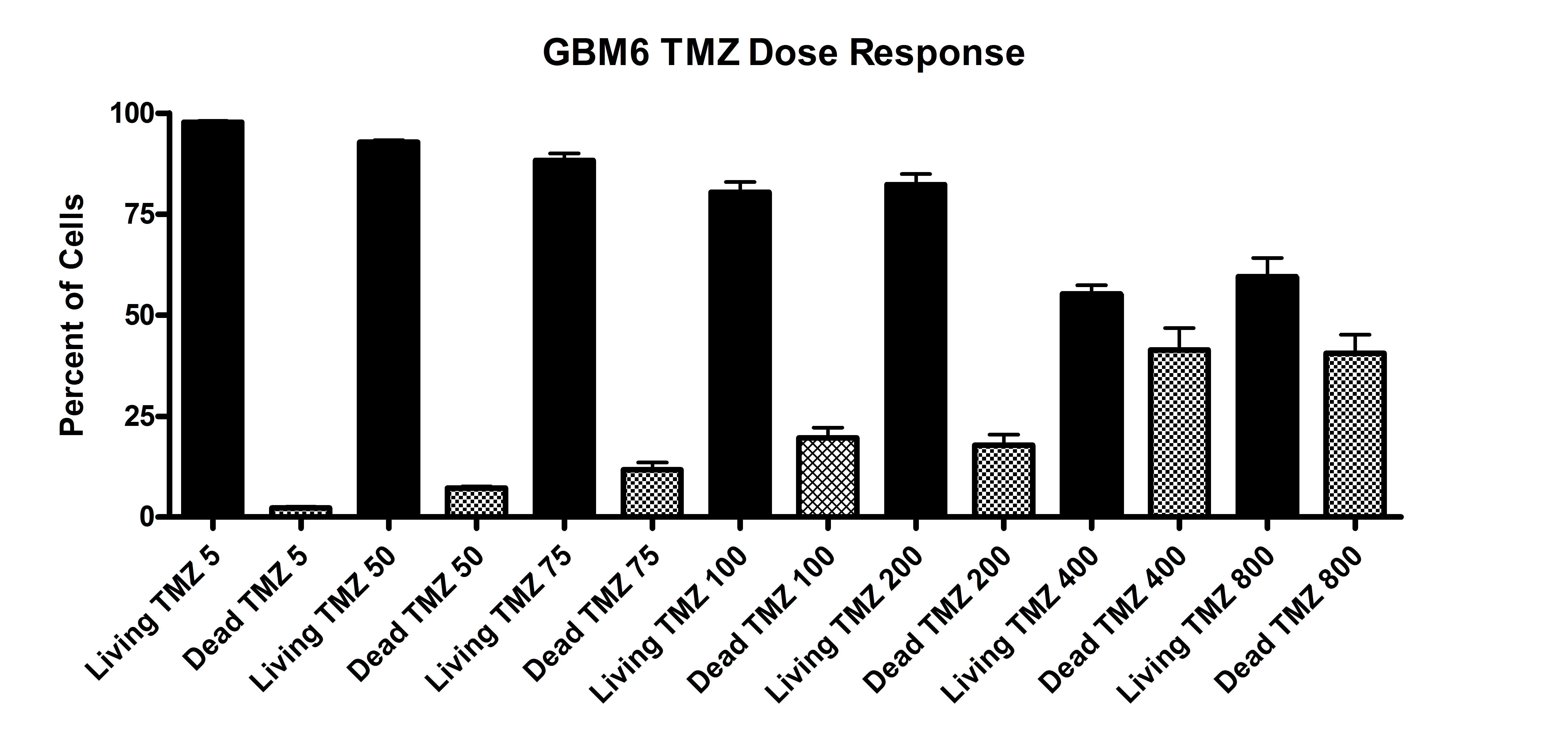


**Supplementary Figure S1:** *Alkylating chemotherapy agent temozolomide induces dose-dependent killing.* PDX GBM 43 cells were treated with TMZ at doses from 5-400μM. After 4 days, both live and dead cells were counted. **Bars** represent means from three independent experiments and **error bars** represent the standard deviation.

**Supplemental Figure S2:** *Bioinformatic analysis of IL-8 expression and Its potential participant in IDH wildtype and mutant Glioblastoma progression and outcomes.* GBM Patients were divided into three groups by their IDH1 status: ***A)*** IDH1 mutant, ***B)*** IDH1 wild type and not applicable. Mutant group and wild type group, respectively, were further stratified into IL8-Up-regulated and IL8-down-regulated groups based on IL8 mRNA expression using quartile (Q1, Q3) as split points. Kaplan-meier survival curves were plotted. Log-rank p value and Wilcoxon p value were evaluated for both groups and Renyi statistics and approximate p value was calculated using SAS for crossed hazard. ***C)*** IDH1 mutant*,* ***D)*** IDH1 wild type and not applicable. Mutant group and wild type group, respectively, were further stratified into IL8-Up-regulated and IL8-down-regulated groups based on IL8 mRNA expression using median as split points. Kaplan-meier survival curves were plotted. Log-rank p value and Wilcoxon p value and Renyi statistics and approximate p value were calculated for both groups.

**
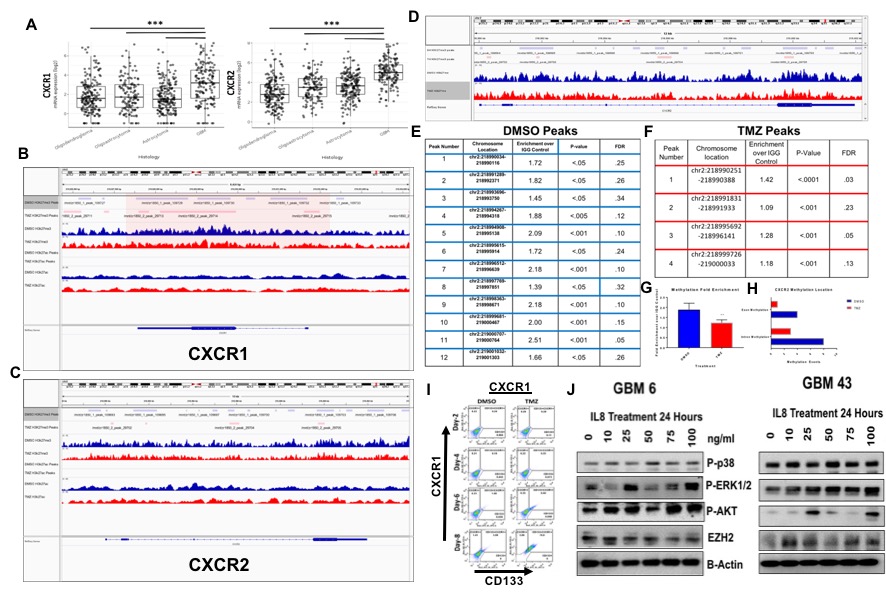
**

**Supplemental Figure S3:** *Therapeutic stress alters the epigenetic regulation of IL-8 receptors CXCR1 and CXCR2 and increases the expression of GIC makers and signaling hubs.* ***A)*** The Cancer Genome Atlas Glioma dataset was examined for expression of IL-8’s main receptors, CXCR1 and CXCR2. Expression of both receptors positively correlated with tumor grade. Experession was compared using one-way ANOVAs with multiple comparisons. ***P<.001. ***B-F)*** CHiPseq analysis of H3K27 acetylation (H3K27ac) and tri-methylation (H3K27me) following treatment with TMZ. Chemotherapy altered the location and levels of H3K27 modification. ***G)*** Total methylation of the CXCR2 Gene was reduced following chemotherapy. *P<.05 based on student t-Test. ***H)*** This reduction was similar in magnitude in both intronic and exonic regions of the gene. ***I)*** FACS analysis of CXCR1 and GIC-marker CD133 following treatment with TMZ demonstrates that therapeutic stress leads to the creation of a CXCR1/CD133 double positive population. ***J)*** Levels of several key signaling nodes were determined following 24 hour exposure to IL-8 in two PDX GBM lines. Specifically, we observed a dose-dependent increase in pERK and p-AKT following IL-8 treatment. Beta-actin served as a loading control.

**
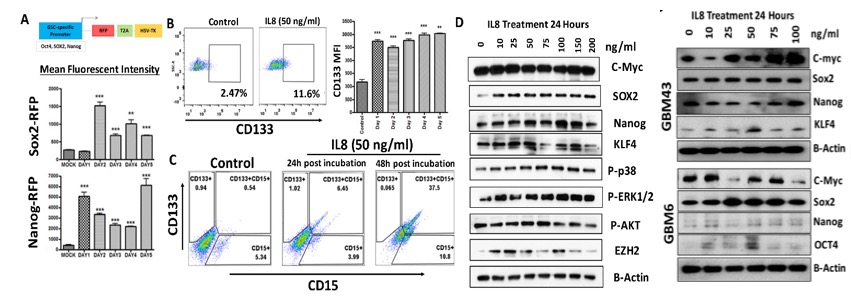
**

**Supplemental Figure S4:** *IL-8 induces the expression of several regulators and markers of the GIC phenotype*. ***A)*** Schematic representation of our construct for high-fidelity and real-time monitoring of the GIC state. Expression of RFP in glioma cells is under the control of a promoter for either SOX2 or NANOG, two key markers of GICs. RFP levels were determined by FACS following treatment with 50ng/mL IL-8 across 5 consecutive days. We observed time-dependent and significant increases in RFP, indicating the induction of a GIC state. ***B)*** PDX GBM43 cells were treated with IL-8 and expression of CD133, a canonical GIC marker, was determined by FACS. Again, we observed the induction of the GIC marker following IL-8 treatment. ***C)*** The GIC induction was measured by the co-expression of CD133 and CD15, another key GIC marker. IL-8 treatment led to a 40% increase in the CD133^+^CD15^+^ GIC population. ***D-E)*** PDX GBM cells were treated with IL-8 at the indicated doses. After 24 hours, expression of many GIC markers were examined by Western blot analysis. Critically, these blots show that IL-8 exposure causes increases in many GIC markers. Bars represent means from two experiments in triplicate and error bars represent the standard deviation. All values were compared to DMSO treated control using student t-Tests. ***P<.001 and ****P<.0001.

**
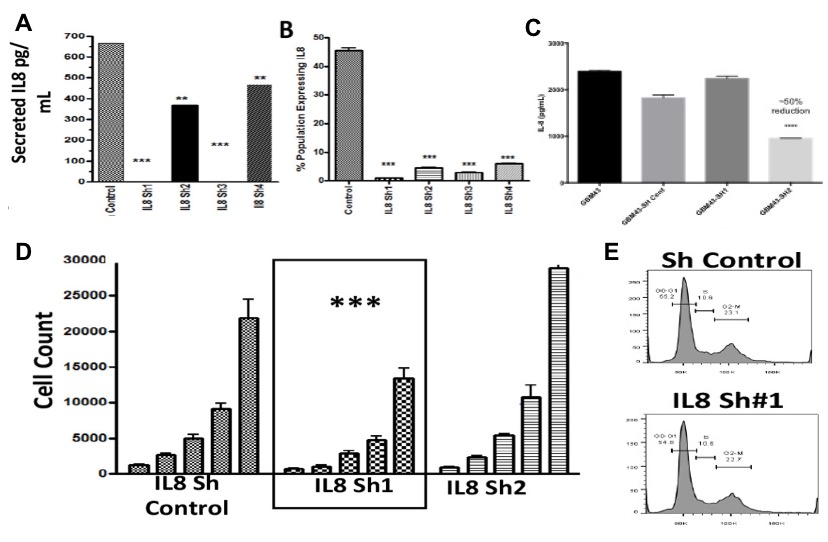
**

**Supplemental Figure S5:** *Knock down of IL-8 by short hairpin RNA reduces IL-8 secretion*. ***A)*** ELISA was used to determine the efficacy of shRNA against IL-8 in U251 cells. Levels were determined 72 hours after plating of purified cells. All four shRNA constructs reduced the secretion of IL8, with varying intensities. ***B)*** IL-8 protein levels in U251 cells expressing shRNA constructs were determined by FACS analysis. All four constructs successfully attenuated IL-8 levels. ***C)*** PDX GBM43 cells were transfected with shRNA against IL-8. ELISA showed that only shRNA construct #2 successfully reduced IL-8 secretion. ***D-E)*** FACS was used to analyze the cell cycle of cells expressing anti IL-8 shRNA. shRNA #1 clearly dysregulates the cell cycle and was therefore excluded from *in vivo* experiments. Bars represent means from two experiments in triplicate and error bars represent the standard deviation. All values were compared to scrambled control shRNA constructs using student t-Tests. **P<.01 ***P<.001 and ****P<.0001.

**
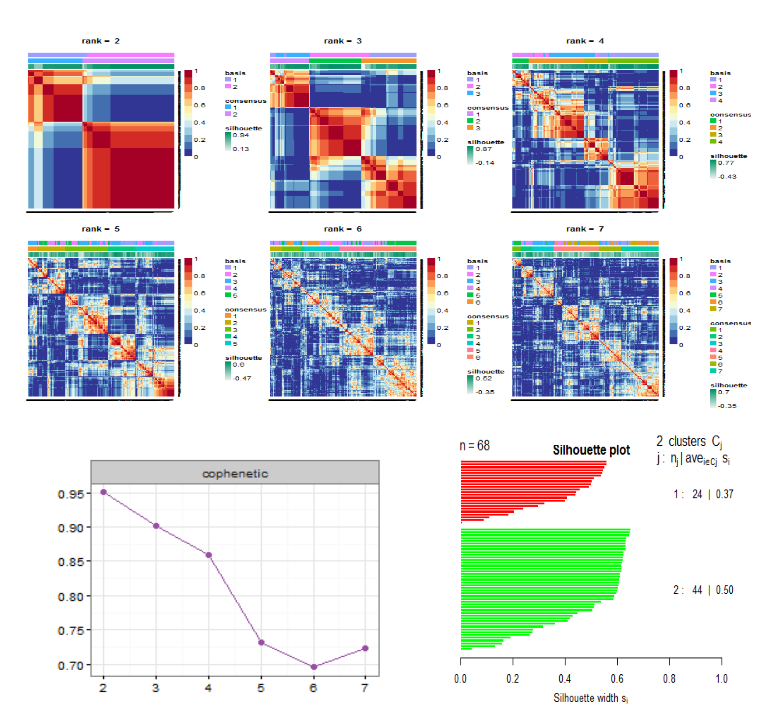
**

**Supplementary Figure S6:** The correlation between IL-8 and all other genes was determined by Pearson correlation coefficients in the TCGA database. Those genes with coefficients >0.5 or <-0.5 and false discovery rate (FDR) <0.05 were selected to be correlated with IL-8. Then Non-negative matrix factorization (NMF) was employed to identify clusters of all the genes that correlate with IL-8 using the R package ‘NMF’ [Reference see below]. Brunet algorithm was used to estimate the factorization. We performed 40 runs for each value of the factorization rank r in range 2:7 to build consensus map. The optimal clusters were determined by the observed cophenetic correlation between clusters and validated by silhouette plot and principle component analysis (PCA). Function “heatmap” was used for plotting the heatmap and clustering, with “euclidean” as the distance measure and “complete” as the clustering method.

**
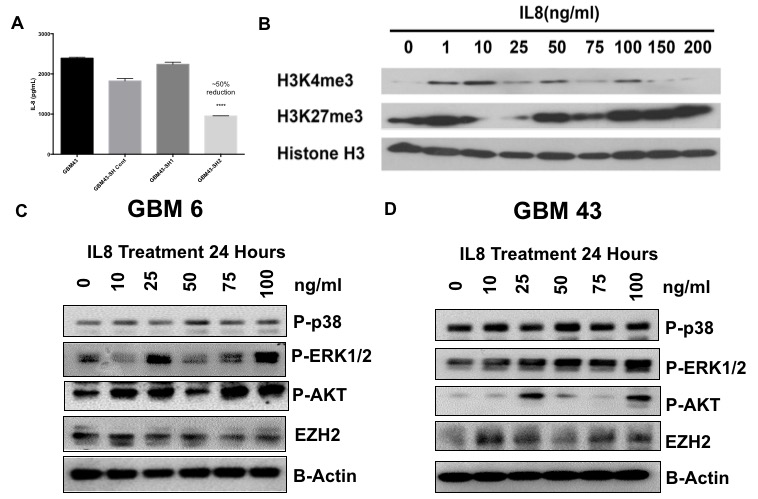
**

**Supplementary Figure S7:** Dose-dependent response to IL-8 in various PDX lines. ***A)*** IL-8 expression from the cell supernatants of GMB43 PDX line transiently infected with lentivirus carrying shRNA against IL-8 transcript. This knockdown line was used in tumor engraftment experiment as described in Figure 5. All data are expressed as the mean fluorescent intensity (MFI). Bars represent means from three independent experiments and error bars represent the standard deviation. One way ANOVAs with multiple comparisons were performed.. **P<.01, ***P<.001. B) GBM43 PDX line was exposed to increasing doses of IL-8 for 72 hours and evaluated for total corresponding histone marks immunoblot analysis. The effect of increasing IL-8 exposure dose on the classical PDX GBM6 (C) and proneural PDX GBM43 (D) was investigated by immunoblot analysis. Representative immunoblot analysis was performed at 72 hours post IL-8 exposure.

**Supplementary Table 1:** Top 300 differentially expressed gene list (fold change) in TMZ treated GBM43.

| **Feature** | **Gene Symbol** | **T-Test** | **Feature P** | **FDR(BH)** | **Fold Change ⇓** | **TMZ8 Mean** | **TMZ8 Std** | **DMSO8 Mean** | **DMSO8 Std** |
| --- | --- | --- | --- | --- | --- | --- | --- | --- | --- |
|  |  |  |  |  |  |  |  |  |  |
| 2260196 | LOC392437 | 34.13816729 | 6.72E-04 | 0.027809405 | 4161.366667 | 6079.9 | 30.48409421 | 1918.533333 | 208.9208542 |
| 2750685 | LOC392437 | 16.3905389 | 0.001230431 | 0.034785259 | 4154.366667 | 5346.3 | 412.0127061 | 1191.933333 | 151.569665 |
| 1230767 | IFITM2 | 23.15064154 | 2.40E-05 | 0.008916308 | 3354.8 | 8945.633333 | 164.9027693 | 5590.833333 | 189.2229461 |
| 6110630 | HIST1H2BK | -23.80197062 | 2.30E-04 | 0.019095488 | 3318.7 | 1199.133333 | 102.9653502 | 4517.833333 | 218.4490864 |
| 7000274 | LOC644039 | 7.188663506 | 0.00935939 | 0.090116029 | 2961.8 | 5850.4 | 668.4820716 | 2888.6 | 249.7759196 |
| 6650242 | IFITM3 | 14.98988022 | 0.002066773 | 0.043526633 | 2881.733333 | 6828.333333 | 98.40387865 | 3946.6 | 318.1059415 |
| 1820504 | NME1 | -10.3544068 | 9.09E-04 | 0.031350327 | 2834.533333 | 3569.1 | 267.555284 | 6403.633333 | 391.4507036 |
| 6860202 | TKT | 37.85883749 | 3.01E-06 | 0.006728593 | 2825.6 | 7024.333333 | 94.08997467 | 4198.733333 | 88.64701537 |
| 5290025 | C14ORF156 | -13.59942695 | 0.003232105 | 0.05366765 | 2796.666667 | 5717.833333 | 88.65925408 | 8514.5 | 344.9786515 |
| 2140128 | SCD | 27.90552214 | 5.34E-04 | 0.025119003 | 2673.466667 | 5427.166667 | 159.3847651 | 2753.7 | 46.1718529 |
| 2340400 | LOC284393 | 10.95203744 | 0.002488599 | 0.04739438 | 2468.533333 | 7961.266667 | 155.0762823 | 5492.733333 | 358.2735314 |
| 2900594 | PGD | 23.03765068 | 0.001331429 | 0.035570432 | 2352.566667 | 3411.433333 | 173.8598957 | 1058.866667 | 32.51466336 |
| 3060377 | MFAP4 | 33.37718186 | 9.48E-06 | 0.007693729 | 2164.3 | 3589.833333 | 89.60744017 | 1425.533333 | 67.70984665 |
| 2940168 | SNRPG | -6.63010898 | 0.008587701 | 0.086594704 | 2158.433333 | 4778.733333 | 234.9932836 | 6937.166667 | 512.568984 |
| 7550458 | RPL12 | 9.085690089 | 0.002810641 | 0.050239008 | 2108.8 | 6081.466667 | 184.9462174 | 3972.666667 | 356.942381 |
| 770326 | LDHA | -11.89836914 | 6.60E-04 | 0.02774538 | 2089.833333 | 3461.966667 | 165.3706544 | 5551.8 | 255.3450802 |
| 1710369 | RPL3 | 14.86681065 | 0.003248982 | 0.05373028 | 2083.433333 | 7477.133333 | 47.63038246 | 5393.7 | 238.0103359 |
| 3830131 | TUBA1C | -4.313146606 | 0.041391762 | 0.208852493 | 2073.9 | 5079.366667 | 188.0027748 | 7153.266667 | 811.3284929 |
| 3610309 | LOC653881 | 4.669823115 | 0.026322961 | 0.159266416 | 1911.6 | 8172.433333 | 248.8385085 | 6260.833333 | 663.9170305 |
| 2600373 | LOC100132795 | 14.52246053 | 3.80E-04 | 0.022589062 | 1882.233333 | 3488.166667 | 119.0331606 | 1605.933333 | 190.3316667 |
| 5270730 | ACTB | -3.559808923 | 0.047608837 | 0.227503144 | 1854.833333 | 4921.5 | 328.0010366 | 6776.333333 | 840.7675917 |
| 3890349 | HIST1H4C | 4.151417447 | 0.043186917 | 0.214569548 | 1835 | 6985.333333 | 188.8512731 | 5150.333333 | 741.9394472 |
| 2710292 | H2AFZ | -27.90011143 | 0.001205619 | 0.034765797 | 1831.4 | 4542.966667 | 113.368176 | 6374.366667 | 8.602519011 |
| 4730025 | LOC341457 | -4.320499546 | 0.022460892 | 0.145081882 | 1817.933333 | 10889.9 | 338.8675995 | 12707.83333 | 645.2201356 |
| 4920767 | FTL | 4.161584894 | 0.022186198 | 0.144330781 | 1809.366667 | 12180.7 | 377.0073872 | 10371.33333 | 651.8909827 |
| 2900390 | VCAM1 | 28.95914781 | 7.12E-04 | 0.028492656 | 1791.166667 | 3186.3 | 104.6468824 | 1395.133333 | 22.93149217 |
| 6770554 | LOC730187 | -6.444466147 | 0.013648849 | 0.110242113 | 1773.233333 | 5094.866667 | 152.2854666 | 6868.1 | 451.5988928 |
| 2340471 | LOC642741 | 13.68219718 | 2.32E-04 | 0.019108789 | 1754.133333 | 6262.533333 | 174.7534358 | 4508.4 | 137.0080655 |
| 1470195 | MCM7 | -39.86169399 | 8.45E-06 | 0.007693729 | 1753.1 | 2742.066667 | 63.04889637 | 4495.166667 | 42.74860622 |
| 4540021 | ATP5B | -12.97715871 | 0.001225944 | 0.034785259 | 1753.033333 | 7829.433333 | 101.0675682 | 9582.466667 | 211.0215234 |
| 4560110 | ARMET | -12.54858218 | 8.85E-04 | 0.030977542 | 1740.533333 | 2571.8 | 209.9706646 | 4312.333333 | 116.7406242 |
| 3390170 | LOC648771 | 10.91793993 | 0.001015848 | 0.032607272 | 1732.866667 | 4027.233333 | 144.2166541 | 2294.366667 | 234.0409437 |
| 6420369 | MDH2 | -6.251108886 | 0.004674947 | 0.063835627 | 1732.7 | 6043.4 | 278.0338828 | 7776.1 | 391.3925268 |
| 3190092 | LDHA | -13.7329049 | 4.89E-04 | 0.024537223 | 1724.3 | 2204.366667 | 113.6243959 | 3928.666667 | 185.4325843 |
| 4780615 | ANXA2 | -12.53752056 | 0.002956654 | 0.051430569 | 1718.966667 | 4199.033333 | 73.15027911 | 5918 | 225.9270457 |
| 5910484 | LOC729742 | 9.617782584 | 8.19E-04 | 0.029829 | 1711.333333 | 3794.666667 | 240.6145119 | 2083.333333 | 192.5785381 |
| 3800168 | SLC2A3 | 14.74700924 | 0.003708599 | 0.057124561 | 1709.866667 | 2703.4 | 198.2971255 | 993.5333333 | 31.76701644 |
| 5570132 | ACTB | -5.438134234 | 0.005615199 | 0.069694256 | 1708.233333 | 3366.8 | 396.8063886 | 5075.033333 | 372.237966 |
| 1450193 | LGALS1 | -37.00947681 | 5.00E-06 | 0.007421726 | 1706.533333 | 3508.466667 | 62.25932326 | 5215 | 50.02369439 |
| 2320731 | LOC729324 | 5.044039055 | 0.014951777 | 0.115404303 | 1706.066667 | 6697.366667 | 519.8731993 | 4991.3 | 270.0722866 |
| 4290358 | CCT7 | -16.79368875 | 4.83E-04 | 0.024485586 | 1672.466667 | 4005.166667 | 153.6741466 | 5677.633333 | 78.34643153 |
| 5340187 | TUBA1A | -10.1432815 | 6.88E-04 | 0.028151784 | 1663.9 | 6572.666667 | 222.7990649 | 8236.566667 | 176.3166564 |
| 6860192 | RPL12 | 11.02245621 | 4.19E-04 | 0.023484716 | 1648.933333 | 8744.133333 | 194.4077245 | 7095.2 | 171.3008757 |
| 830593 | VIM | -6.503353559 | 0.00337564 | 0.054717492 | 1641.166667 | 7948.866667 | 340.8646701 | 9590.033333 | 273.6117931 |
| 4860719 | ROCK2 | 3.38133448 | 0.031894264 | 0.179221524 | 1633.966667 | 6836.133333 | 492.4913231 | 5202.166667 | 676.7494169 |
| 70605 | HSPD1 | -5.320068458 | 0.030091147 | 0.173027898 | 1633.033333 | 4716.7 | 84.3461321 | 6349.733333 | 524.9322845 |
| 5810328 | FTL | 4.894097166 | 0.008126633 | 0.084062858 | 1632.566667 | 12166.93333 | 418.3285352 | 10534.36667 | 398.5292001 |
| 5090326 | LOC100132795 | 11.37811246 | 0.004826388 | 0.064325553 | 1607.033333 | 3466.833333 | 236.9642659 | 1859.8 | 60.77260896 |
| 1570059 | LOC100133649 | 4.135322464 | 0.026277579 | 0.159171712 | 1599.166667 | 4768.3 | 597.4742421 | 3169.133333 | 302.7477883 |
| 1410021 | AIF1L | -23.63781464 | 1.95E-05 | 0.008810364 | 1592.4 | 3102.6 | 80.04217638 | 4695 | 84.90017668 |
| 7100711 | CALM2 | -6.993812637 | 0.016625192 | 0.122200453 | 1577.433333 | 2549.8 | 70.75471716 | 4127.233333 | 384.1979872 |
| 5810605 | CCNI | 11.40784451 | 0.003784784 | 0.057665621 | 1573.933333 | 4220.466667 | 72.53146444 | 2646.533333 | 227.6968233 |
| 4250445 | RPL4 | 10.66006505 | 0.003127127 | 0.052993929 | 1572.066667 | 4586.2 | 94.80606521 | 3014.133333 | 237.1839862 |
| 5550463 | FARSLB | 3.323230288 | 0.057271365 | 0.253435967 | 1566 | 6191.333333 | 765.428392 | 4625.333333 | 283.3506191 |
| 2100112 | LOC439953 | -4.298837035 | 0.013771459 | 0.110794562 | 1562.233333 | 8233.833333 | 398.2042483 | 9796.066667 | 487.4733771 |
| 1090500 | LOC645166 | -24.34963117 | 0.001657641 | 0.039180943 | 1549 | 343.0333333 | 4.201587002 | 1892.033333 | 110.1041477 |
| 1440736 | LDLR | 34.92009997 | 1.60E-05 | 0.008416524 | 1542.6 | 2337.166667 | 64.00549456 | 794.5666667 | 41.92401857 |
| 6480468 | BEX1 | -14.0414373 | 0.004880319 | 0.064679954 | 1539.133333 | 449.3666667 | 11.80225967 | 1988.5 | 189.4892345 |
| 2320634 | LOC389342 | 6.925541732 | 0.002329892 | 0.04620168 | 1538.366667 | 9783.933333 | 282.1265201 | 8245.566667 | 261.5895322 |
| 5890528 | PPIAL4A | -7.856068906 | 0.001420886 | 0.036524723 | 1536.3 | 8581.066667 | 241.9310301 | 10117.36667 | 237.0561396 |
| 5810129 | RPS8 | 6.293536619 | 0.012753317 | 0.106346567 | 1532.866667 | 5472.5 | 394.5316337 | 3939.633333 | 149.3726994 |
| 620280 | JUND | 9.264473312 | 0.007471591 | 0.080401831 | 1523.5 | 5472.033333 | 72.44765927 | 3948.533333 | 275.459985 |
| 4220181 | DAD1 | -10.28727968 | 0.007135989 | 0.078300594 | 1523.266667 | 2725 | 50.31937996 | 4248.266667 | 251.4848968 |
| 1690608 | ALDH3A2 | 38.02729091 | 1.36E-05 | 0.007826421 | 1520.066667 | 2550.166667 | 37.30554025 | 1030.1 | 58.32520896 |
| 1170706 | GABPB2 | 6.212117485 | 0.004843338 | 0.064442551 | 1518.1 | 4104.333333 | 346.0481662 | 2586.233333 | 243.7444222 |
| 6550386 | HS.508682 | 10.56587376 | 0.001275136 | 0.035076626 | 1510.333333 | 5970.133333 | 125.8288653 | 4459.8 | 213.2286801 |
| 5820129 | PABPC1 | -9.618041593 | 0.001182144 | 0.034586528 | 1510.266667 | 6271.933333 | 153.0727387 | 7782.2 | 224.8080737 |
| 4730181 | FTHL12 | 9.41871648 | 0.005969645 | 0.071833113 | 1497.366667 | 2852.033333 | 262.4367416 | 1354.666667 | 83.35924264 |
| 3170286 | LOC647000 | -7.555627654 | 0.001644355 | 0.039094262 | 1493.633333 | 4091.1 | 241.7601911 | 5584.733333 | 242.4663757 |
| 4280113 | NFKBIA | 11.69972443 | 3.68E-04 | 0.022383175 | 1487.7 | 3528.666667 | 140.6014343 | 2040.966667 | 169.5223387 |
| 1580025 | GNG11 | -17.91639366 | 0.001988088 | 0.042616251 | 1480.066667 | 1888.2 | 31.3702088 | 3368.266667 | 139.6028772 |
| 6350189 | MGC4677 | -8.445883882 | 0.007830479 | 0.08249639 | 1478.5 | 1291.8 | 90.58018547 | 2770.3 | 289.3591367 |
| 1070215 | CAV1 | -5.627675878 | 0.018535104 | 0.129959972 | 1473.133333 | 1974.866667 | 146.2711295 | 3448 | 429.1489602 |
| 3850762 | LOC649076 | -5.038595834 | 0.034656518 | 0.188259299 | 1462.833333 | 8515.6 | 498.517442 | 9978.433333 | 65.93340074 |
| 4230520 | DNCL1 | -7.182906356 | 0.016040739 | 0.119727324 | 1459.033333 | 2756.033333 | 60.23996459 | 4215.066667 | 346.6285812 |
| 7650433 | TIMP1 | 10.96990728 | 4.10E-04 | 0.023414414 | 1452.3 | 4476.7 | 154.542842 | 3024.4 | 169.4030991 |
| 520209 | SRP9 | -5.04059511 | 0.019257125 | 0.133128526 | 1437.766667 | 2801.566667 | 193.6633505 | 4239.333333 | 454.5060762 |
| 4150670 | CDC37 | -6.937361612 | 0.006207838 | 0.073027761 | 1426.766667 | 3549.833333 | 163.0187821 | 4976.6 | 316.7303585 |
| 3140709 | LOC100131196 | 4.224466549 | 0.047074098 | 0.225888831 | 1423.933333 | 4576.433333 | 95.51095923 | 3152.5 | 575.9536179 |
| 1070047 | CDC42EP4 | -8.163953335 | 0.011220067 | 0.099134683 | 1423.733333 | 2536.5 | 63.74896078 | 3960.233333 | 295.2531851 |
| 3840598 | LOC643531 | 6.659402753 | 0.003744754 | 0.057395294 | 1421.766667 | 6089.133333 | 300.983211 | 4667.366667 | 214.8320584 |
| 3890630 | LOC727808 | 3.023249991 | 0.039094369 | 0.201945874 | 1415.566667 | 6194.133333 | 582.9291238 | 4778.566667 | 563.8285585 |
| 20446 | CEBPB | 8.759856887 | 0.006018744 | 0.071968301 | 1410.133333 | 4814.266667 | 94.8153117 | 3404.133333 | 262.2033626 |
| 4260139 | AKR1B1 | -4.615209861 | 0.043606062 | 0.21585894 | 1409.4 | 1378.066667 | 21.52796631 | 2787.466667 | 528.4981015 |
| 4890615 | RPL6 | -7.72150071 | 0.001980022 | 0.042556483 | 1375.533333 | 6455.233333 | 187.3005695 | 7830.766667 | 245.2010672 |
| 290603 | AARS | 38.81972771 | 8.83E-05 | 0.013558219 | 1373.733333 | 2957.966667 | 23.87976828 | 1584.233333 | 56.44983023 |
| 360192 | INSIG1 | 38.03749657 | 4.92E-05 | 0.010760817 | 1358.033333 | 1855.633333 | 55.42358102 | 497.6 | 27.4268117 |
| 7210026 | LOC100131713 | 5.454729262 | 0.007161612 | 0.078467443 | 1353.166667 | 5445.566667 | 348.1484214 | 4092.4 | 251.8176324 |
| 360047 | GAS1 | 23.67939155 | 2.85E-05 | 0.009573064 | 1352.466667 | 1847.4 | 77.50270963 | 494.9333333 | 61.4814064 |
| 270397 | LOC100130003 | -45.93353008 | 3.27E-06 | 0.006728593 | 1334.1 | 3565.866667 | 29.85738323 | 4899.966667 | 40.48732312 |
| 2690047 | ARL6IP1 | -38.26078054 | 4.18E-04 | 0.023484716 | 1328.533333 | 1405.066667 | 11.98012243 | 2733.6 | 58.93691543 |
| 1170609 | ITIH5 | 2.492201435 | 0.070584255 | 0.286612919 | 1315.9 | 8282.433333 | 568.0231715 | 6966.533333 | 716.7454941 |
| 6900608 | LOC641814 | -3.810167375 | 0.061883426 | 0.265357296 | 1315.833333 | 6681.266667 | 597.2254544 | 7997.1 | 33.42588817 |
| 5080192 | SERPINE2 | -10.28116688 | 0.003024616 | 0.052105159 | 1314.6 | 5550.5 | 87.01517109 | 6865.1 | 203.6581204 |
| 4120307 | NEDD8 | -6.411869711 | 0.014667132 | 0.114345143 | 1311.4 | 4943.1 | 106.7895126 | 6254.5 | 337.7718017 |
| 2320079 | NME4 | 17.34858175 | 7.65E-04 | 0.029202808 | 1300.866667 | 2512.433333 | 119.5960423 | 1211.566667 | 50.64191281 |
| 1500707 | LOC729603 | 5.063453018 | 0.018150466 | 0.128500847 | 1299.733333 | 3459.933333 | 406.3469618 | 2160.2 | 180.4163795 |
| 2370114 | LOC441013 | 5.804737636 | 0.010154858 | 0.094283153 | 1295.633333 | 6517.066667 | 177.8997002 | 5221.433333 | 343.234735 |
| 3610717 | ZNF674 | 2.744484295 | 0.055027102 | 0.247558365 | 1280.266667 | 8019.666667 | 497.1906107 | 6739.4 | 636.8926833 |
| 3850204 | FTHL16 | 6.018893276 | 0.00969569 | 0.091661456 | 1277.6 | 11391.2 | 164.6600437 | 10113.6 | 328.7191506 |
| 6510072 | ACTG1 | -4.019895201 | 0.051356459 | 0.238588419 | 1275.8 | 4035.866667 | 94.21148196 | 5311.666667 | 541.5700539 |
| 3520168 | HS.579631 | -12.78312602 | 3.92E-04 | 0.022901887 | 1271.033333 | 840.9333333 | 99.64016928 | 2111.966667 | 140.4675882 |
| 1780543 | PSMB7 | -7.06339957 | 0.002135489 | 0.044012694 | 1270.833333 | 3432.8 | 215.3269375 | 4703.633333 | 225.2684251 |
| 5860347 | AKR1D1 | 5.406730674 | 0.005667126 | 0.070045558 | 1270.633333 | 4127.1 | 287.1470181 | 2856.466667 | 288.5048584 |
| 5490431 | SAT1 | 22.83258371 | 4.68E-05 | 0.010749592 | 1270.566667 | 2021.566667 | 78.24450992 | 751 | 56.28134682 |
| 780170 | LOC100128505 | 3.138905995 | 0.046726106 | 0.224857503 | 1262.533333 | 3919.766667 | 352.6658664 | 2657.233333 | 600.8090739 |
| 2260095 | EIF4G2 | -11.59412186 | 3.20E-04 | 0.02108965 | 1258.766667 | 2359.8 | 130.0494906 | 3618.566667 | 135.8273291 |
| 2640091 | GAPDH | -2.765431431 | 0.09164898 | 0.334460457 | 1256.3 | 4745.4 | 225.1459749 | 6001.7 | 753.9494479 |
| 7380349 | LILRB3 | 4.139314855 | 0.020043089 | 0.136283291 | 1253.866667 | 3863.766667 | 442.2836345 | 2609.9 | 282.2416695 |
| 4230050 | TIMM23 | -11.28757133 | 0.002169572 | 0.044413513 | 1251.2 | 2985.9 | 77.9319575 | 4237.1 | 175.4655807 |
| 6940066 | PFN1 | -5.891498975 | 0.00869708 | 0.08707017 | 1244.7 | 6768.033333 | 319.9717852 | 8012.733333 | 177.5492702 |
| 6380255 | RPS3A | 8.976059985 | 0.00834782 | 0.085214405 | 1236.166667 | 5051.566667 | 57.75823174 | 3815.4 | 231.4365572 |
| 5670465 | ADM | -18.34335575 | 0.001973231 | 0.042475641 | 1225.8 | 1378.866667 | 24.13489866 | 2604.666667 | 113.2005448 |
| 1500010 | CDC20 | -21.86491415 | 3.97E-05 | 0.010275357 | 1222.533333 | 817.5333333 | 59.84098373 | 2040.066667 | 76.1436362 |
| 2850592 | LOC728937 | -3.494801839 | 0.05868019 | 0.257064029 | 1217.3 | 8036.3 | 165.2036017 | 9253.6 | 580.2435954 |
| 5700142 | FDFT1 | 12.15295833 | 6.01E-04 | 0.026319406 | 1216.5 | 3350 | 94.7332571 | 2133.5 | 145.2070591 |
| 3130736 | LOC645688 | 10.95531121 | 5.59E-04 | 0.025609591 | 1207 | 2683.633333 | 151.5893906 | 1476.633333 | 115.9144656 |
| 4730392 | MYL12A | -2.991913432 | 0.08389279 | 0.317087672 | 1205.833333 | 2052.666667 | 164.1472002 | 3258.5 | 678.4963007 |
| 6290367 | SEC61G | -6.877121073 | 0.005992217 | 0.071899844 | 1202.8 | 4383.566667 | 142.1784911 | 5586.366667 | 267.4958193 |
| 770689 | LOC100133233 | 6.07773045 | 0.012691214 | 0.106005721 | 1199.333333 | 2894.766667 | 316.4508229 | 1695.433333 | 129.1477578 |
| 4610390 | ACTB | -3.941514629 | 0.051646172 | 0.239285837 | 1192.6 | 4941.266667 | 103.6475438 | 6133.866667 | 513.722039 |
| 6180066 | NACA | 5.446000543 | 0.024259688 | 0.151958234 | 1184.8 | 4131.6 | 94.12486388 | 2946.8 | 364.8697439 |
| 5310634 | FASN | 6.06461357 | 0.016225161 | 0.120611738 | 1175.766667 | 3864.466667 | 104.0714338 | 2688.7 | 319.2643262 |
| 3890326 | SOD2 | 6.343217907 | 0.004776551 | 0.064101107 | 1173.533333 | 3634.333333 | 180.6245369 | 2460.8 | 264.6817523 |
| 3460477 | H3F3A | -3.723797581 | 0.020612097 | 0.138312403 | 1173.166667 | 5678.766667 | 400.0712603 | 6851.933333 | 371.0855607 |
| 5900379 | SRP14 | -7.873269629 | 0.002584571 | 0.048201174 | 1172.933333 | 3106.233333 | 140.6421108 | 4279.166667 | 216.3375218 |
| 3850131 | YWHAQ | -6.213534366 | 0.007224429 | 0.07883094 | 1172.666667 | 3499.066667 | 284.6994965 | 4671.733333 | 160.6260979 |
| 2340035 | LOC644464 | -3.13729645 | 0.075176902 | 0.297299756 | 1160.133333 | 5087.666667 | 160.5822323 | 6247.8 | 620.0338378 |
| 3390603 | MDH1 | -11.33217772 | 5.52E-04 | 0.025451648 | 1153.3 | 2866.466667 | 142.2673657 | 4019.766667 | 104.0802735 |
| 5220296 | CCR6 | 3.246571219 | 0.031853817 | 0.179079311 | 1149.266667 | 7221 | 413.4180814 | 6071.733333 | 452.7921193 |
| 1770102 | NDUFB3 | -14.57601749 | 1.65E-04 | 0.016596044 | 1146.866667 | 2288.266667 | 86.21973865 | 3435.133333 | 105.5396292 |
| 3780717 | SRXN1 | 28.02599823 | 2.34E-04 | 0.019213523 | 1144.533333 | 1385.033333 | 65.44496416 | 240.5 | 26.8374738 |
| 4490706 | BUD31 | -5.670489833 | 0.026110199 | 0.158605467 | 1141.033333 | 1918.9 | 58.60819055 | 3059.933333 | 343.5654862 |
| 1240035 | LOC441073 | 5.181963582 | 0.024784761 | 0.153640081 | 1140.466667 | 3261.033333 | 108.8876638 | 2120.566667 | 365.3138696 |
| 430593 | RPL31 | 5.188652209 | 0.021104278 | 0.140482792 | 1137.8 | 9362.966667 | 356.9052162 | 8225.166667 | 129.9154469 |
| 730414 | APOE | 4.810844243 | 0.035779402 | 0.191544088 | 1134.033333 | 5334.866667 | 72.68083195 | 4200.833333 | 401.7654332 |
| 6520128 | GPX4 | 16.52671645 | 2.79E-04 | 0.020263759 | 1129.133333 | 2946.533333 | 101.2411642 | 1817.4 | 61.26818098 |
| 6580189 | LOC387820 | 13.88047813 | 0.001028978 | 0.032718471 | 1127.433333 | 3565.833333 | 127.0170592 | 2438.4 | 60.48859397 |
| 3940446 | TXN | 3.944370637 | 0.016929243 | 0.123305141 | 1122.566667 | 10039.8 | 353.9411957 | 8917.233333 | 343.0985038 |
| 6760669 | SPRY1 | 17.70648046 | 1.43E-04 | 0.015832666 | 1121.866667 | 3065.433333 | 90.71892489 | 1943.566667 | 61.75114034 |
| 2480711 | LOC285176 | 7.449667547 | 0.001745875 | 0.040229493 | 1121.3 | 11042.03333 | 180.5374292 | 9920.733333 | 188.0748344 |
| 4260368 | UBE2C | -32.23235914 | 6.89E-06 | 0.007693729 | 1119.1 | 812.2666667 | 45.67070542 | 1931.366667 | 39.12254252 |
| 6420168 | DBNDD2 | 11.44051243 | 0.001763123 | 0.040355726 | 1116.066667 | 2573.566667 | 72.82014373 | 1457.5 | 152.4713744 |
| 5090561 | ATP5EP2 | -3.756542605 | 0.019981241 | 0.136027687 | 1115.133333 | 6340.4 | 351.7296263 | 7455.533333 | 375.0303632 |
| 6770746 | LOC728715 | 18.34055968 | 0.001106439 | 0.033913719 | 1112.666667 | 1704.666667 | 99.57672084 | 592 | 33.55488042 |
| 7550470 | KIAA0101 | 3.535734431 | 0.045275504 | 0.220635761 | 1111.866667 | 8205.6 | 213.9718907 | 7093.733333 | 500.8809972 |
| 6110672 | ACP1 | -13.25175868 | 0.003149077 | 0.053017712 | 1109.7 | 1862.066667 | 38.91045275 | 2971.766667 | 139.7249202 |
| 1300491 | POLE3 | -27.80748759 | 3.79E-05 | 0.010093194 | 1108.466667 | 1510.166667 | 58.03932575 | 2618.633333 | 37.39536513 |
| 4120086 | LAMC1 | 14.05845922 | 0.003199315 | 0.053337691 | 1105.533333 | 4394.066667 | 31.78825779 | 3288.533333 | 132.4441518 |
| 3610286 | GLTSCR2 | 5.134040108 | 0.016343072 | 0.120991409 | 1100.033333 | 3471.6 | 157.6935953 | 2371.566667 | 335.9438247 |
| 2650619 | HSPA8 | -10.86415207 | 0.00103895 | 0.032880848 | 1099.2 | 3557.833333 | 149.2851745 | 4657.033333 | 91.78324103 |
| 4290543 | TXN | 5.609849607 | 0.008333178 | 0.085156795 | 1094.866667 | 6061.966667 | 178.2825099 | 4967.1 | 287.2067722 |
| 3940370 | RPLP0 | -4.610649792 | 0.023210244 | 0.147921537 | 1092.466667 | 9906.833333 | 165.3575621 | 10999.3 | 375.6121936 |
| 4590670 | LOC646294 | 11.0052145 | 0.002838293 | 0.050466362 | 1090.033333 | 3879.466667 | 159.127915 | 2789.433333 | 64.10322405 |
| 4060358 | ABCA1 | 34.30023196 | 5.31E-06 | 0.00749042 | 1088.6 | 1643.133333 | 41.62707452 | 554.5333333 | 35.90227477 |
| 2370193 | RPS15A | 6.762251917 | 0.007743284 | 0.081924356 | 1087.8 | 3723.166667 | 119.1027428 | 2635.366667 | 251.884504 |
| 650377 | LOC343184 | 2.607973991 | 0.063927069 | 0.270419788 | 1087 | 7738.333333 | 436.1394769 | 6651.333333 | 575.2787701 |
| 5870328 | LOC440589 | 3.130714589 | 0.035435582 | 0.19059488 | 1083.8 | 8762.866667 | 407.6646334 | 7679.066667 | 439.7015389 |
| 160370 | TPM2 | -15.20522205 | 4.25E-04 | 0.023605402 | 1082.366667 | 1914 | 61.83599276 | 2996.366667 | 106.6665052 |
| 2260349 | MIR1974 | -5.105788136 | 0.02007668 | 0.136409587 | 1080.233333 | 6355.933333 | 135.5834184 | 7436.166667 | 340.4455365 |
| 4040671 | COL1A2 | 13.5465423 | 2.13E-04 | 0.018612809 | 1077.766667 | 2456.766667 | 106.2972405 | 1379 | 87.69498275 |
| 2510411 | SLC44A4 | 6.152002671 | 0.017775553 | 0.126795194 | 1074.9 | 2413.8 | 291.5491897 | 1338.9 | 81.14228244 |
| 650301 | SNRPF | -7.672218372 | 0.01144296 | 0.100280089 | 1072.1 | 1751.366667 | 60.96936389 | 2823.466667 | 234.2281224 |
| 6420424 | PAICS | -14.14881536 | 1.52E-04 | 0.016269522 | 1071.5 | 1864.566667 | 96.58448806 | 2936.066667 | 88.75169482 |
| 1340358 | CREB1 | 3.83623301 | 0.018682819 | 0.130569998 | 1070.4 | 2826.666667 | 353.3117085 | 1756.266667 | 329.7476965 |
| 5820601 | CCND1 | -14.02587922 | 0.004895492 | 0.064739529 | 1063.966667 | 1399.033333 | 8.05005176 | 2463 | 131.1420223 |
| 2810050 | LOC390345 | 14.6549487 | 0.002624051 | 0.048475816 | 1063.233333 | 1843.166667 | 121.407427 | 779.9333333 | 32.42288287 |
| 3890398 | WBP2 | 15.85611075 | 0.002971677 | 0.05159704 | 1061 | 3595.866667 | 113.9713268 | 2534.866667 | 21.04978226 |
| 7330689 | LOC728672 | 4.508418148 | 0.011608182 | 0.100980279 | 1060.5 | 3005.4 | 313.4851352 | 1944.9 | 260.2340677 |
| 4540600 | FAM115A | 2.652427547 | 0.056896639 | 0.252509857 | 1057.8 | 4824.6 | 480.6312204 | 3766.8 | 496.1135757 |
| 5050402 | HIST1H2BK | -12.54695302 | 0.005423474 | 0.068381221 | 1057.3 | 232.6 | 20.35313244 | 1289.9 | 144.529478 |
| 4900520 | SCG2 | -21.42145802 | 9.71E-04 | 0.032132852 | 1053.633333 | 336.4666667 | 23.94625928 | 1390.1 | 81.75775193 |
| 2340452 | RN7SL1 | 2.273950616 | 0.094000179 | 0.339609381 | 1053.366667 | 7398.633333 | 453.7799724 | 6345.266667 | 661.6912901 |
| 4860286 | UBB | -2.631291711 | 0.076248678 | 0.299902616 | 1052.066667 | 8485.766667 | 609.3509361 | 9537.833333 | 329.0608809 |
| 940711 | ATP5A1 | -6.825163712 | 0.009499116 | 0.090759531 | 1051.8 | 4290.233333 | 247.2046588 | 5342.033333 | 100.6777698 |
| 450615 | MT2A | -7.078028452 | 0.017947043 | 0.127537759 | 1051.633333 | 680.4 | 30.76930288 | 1732.033333 | 255.4970907 |
| 2000593 | RPL17 | 18.48295282 | 1.77E-04 | 0.017009472 | 1048.466667 | 2492.333333 | 51.74073186 | 1443.866667 | 83.52522573 |
| 7040678 | FABP7 | 4.22539104 | 0.051347237 | 0.238588419 | 1046.133333 | 4281.333333 | 19.11029391 | 3235.2 | 428.3996265 |
| 830326 | LOC400721 | 5.294047137 | 0.023092645 | 0.147410333 | 1046.066667 | 3630.266667 | 327.0542666 | 2584.2 | 100.8190954 |
| 1410750 | EEF1AL7 | 6.84749905 | 0.005793079 | 0.070935235 | 1045.933333 | 3339.3 | 126.5332763 | 2293.366667 | 232.3446865 |
| 3610064 | NACA | 2.706517874 | 0.109573639 | 0.369757305 | 1043.533333 | 8669.466667 | 88.07169428 | 7625.933333 | 661.9818829 |
| 1260161 | LOC100133465 | 2.53003458 | 0.06662747 | 0.277035958 | 1040.6 | 8564.266667 | 456.533595 | 7523.666667 | 546.8793316 |
| 610112 | FAM177A1 | 2.312202275 | 0.08517319 | 0.320111552 | 1038.1 | 7724.666667 | 484.055021 | 6686.566667 | 608.6065259 |
| 4210095 | ATP1A1 | -17.9693259 | 5.88E-04 | 0.026083667 | 1031.4 | 842.8333333 | 40.78508714 | 1874.233333 | 90.66478552 |
| 5490202 | LOC646688 | 3.151611289 | 0.084666056 | 0.319041873 | 1030.766667 | 3098.266667 | 65.80336871 | 2067.5 | 562.6500422 |
| 2480041 | LOC148430 | 3.565994248 | 0.056922821 | 0.252578668 | 1029.266667 | 1537.633333 | 481.7419053 | 508.3666667 | 133.6160295 |
| 380753 | SEC61G | -8.959414508 | 0.003833999 | 0.058042807 | 1029.2 | 5005.266667 | 82.66107508 | 6034.466667 | 180.9833234 |
| 4050435 | LOC645715 | 2.904408099 | 0.044234781 | 0.21758233 | 1028.6 | 5079.5 | 416.8199731 | 4050.9 | 450.0340098 |
| 2120703 | LOC645173 | 5.827095331 | 0.010822838 | 0.097508301 | 1027.766667 | 2508.2 | 273.8634149 | 1480.433333 | 135.3719444 |
| 1940593 | LOC653226 | -4.179904622 | 0.046046194 | 0.222718221 | 1027.366667 | 2388.866667 | 83.91640682 | 3416.233333 | 417.3630833 |
| 630128 | LOC286444 | 5.21055861 | 0.006471119 | 0.07462479 | 1019.966667 | 2351.733333 | 241.0041563 | 1331.766667 | 238.4767144 |
| 2970431 | FTHL7 | 5.280186976 | 0.007988395 | 0.083270088 | 1017.666667 | 9823.466667 | 270.6038494 | 8805.8 | 195.478055 |
| 4640689 | EIF4A2 | 17.12219298 | 0.001758618 | 0.040313593 | 1006.766667 | 2304.2 | 98.13322577 | 1297.433333 | 27.23625035 |
| 6370270 | GJC1 | 3.201019826 | 0.034467287 | 0.187759168 | 1006.366667 | 4159.966667 | 419.0810224 | 3153.6 | 347.696937 |
| 3310424 | ACTG1 | -2.304128146 | 0.109936009 | 0.370572034 | 1006.066667 | 8319.866667 | 685.6364513 | 9325.933333 | 319.1507533 |
| 4180576 | HNRNPD | -6.550858946 | 0.004483721 | 0.062600383 | 1003.6 | 3415 | 220.7690422 | 4418.6 | 147.2172884 |
| 870338 | EGR1 | 8.338319283 | 0.001274167 | 0.035076626 | 1003.366667 | 1725.166667 | 159.2470513 | 721.8 | 134.4609609 |
| 2600113 | LOC389517 | 5.098832225 | 0.011980417 | 0.102603623 | 1002.966667 | 2538.166667 | 293.1658291 | 1535.2 | 173.5873555 |
| 6580605 | LOC646294 | 5.810058698 | 0.007331489 | 0.079414397 | 1001.966667 | 5009.4 | 252.9631594 | 4007.433333 | 158.8413464 |
| 2030484 | GLRX5 | -5.354603508 | 0.023888454 | 0.150476174 | 1001.4 | 2826.5 | 87.98585114 | 3827.9 | 311.743757 |
| 1500189 | CD81 | 3.015686389 | 0.06536551 | 0.273884498 | 1000.933333 | 7840.333333 | 223.8525035 | 6839.4 | 529.509896 |
| 840296 | LOC440926 | -6.922578756 | 0.004327696 | 0.06152662 | 997.7666667 | 5387.433333 | 131.5778223 | 6385.2 | 212.1545899 |
| 6330408 | LOC388339 | 6.465596562 | 0.004224495 | 0.060823053 | 992.1333333 | 2769.3 | 153.2100845 | 1777.166667 | 217.1764337 |
| 4810474 | IL18 | 3.27692609 | 0.061375119 | 0.263799273 | 989.7333333 | 2438 | 494.5834712 | 1448.266667 | 170.4564558 |
| 3190452 | DNAJB11 | -13.05176176 | 3.07E-04 | 0.021036856 | 989.6333333 | 1639.433333 | 79.00008439 | 2629.066667 | 104.9128368 |
| 1170709 | CDH2 | -3.870333486 | 0.052452717 | 0.241573382 | 986.7 | 2092.466667 | 93.95793385 | 3079.166667 | 431.4556795 |
| 4260414 | H2AFY | -5.996403146 | 0.025926362 | 0.157995863 | 979.8666667 | 4344.7 | 22.32420211 | 5324.566667 | 282.1510293 |
| 6060356 | RPL13A | 10.25747791 | 0.002354103 | 0.046467273 | 978.6666667 | 2549.2 | 71.67175176 | 1570.533333 | 148.9040071 |
| 520671 | NDUFB3 | -7.3386697 | 0.010858751 | 0.097693657 | 977 | 2296.833333 | 68.82015209 | 3273.833333 | 220.0792887 |
| 6040609 | PNPT1 | 4.068857883 | 0.01537114 | 0.117010864 | 970.1 | 2375.2 | 301.4806627 | 1405.1 | 282.210418 |
| 1110338 | MYL6 | -19.72876021 | 7.47E-04 | 0.028869031 | 968.1 | 2642.2 | 79.62920319 | 3610.3 | 29.71413805 |
| 1510296 | ASNS | 8.128919233 | 0.007663119 | 0.081482494 | 967.0666667 | 2663.6 | 67.25674687 | 1696.533333 | 194.7701295 |
| 3360113 | MEG3 | 4.969342564 | 0.028610679 | 0.1672826 | 967 | 2675.933333 | 88.86311571 | 1708.933333 | 325.1197062 |
| 1190367 | IER3 | 6.903018529 | 0.009807892 | 0.092348375 | 960.3 | 2690.566667 | 87.43570971 | 1730.266667 | 224.5268878 |
| 4260112 | LOC100133177 | 3.203015804 | 0.038121701 | 0.199060829 | 957.0666667 | 2922.4 | 422.7270159 | 1965.333333 | 298.5787892 |
| 4210403 | SLC1A3 | 13.46964255 | 0.001079597 | 0.033567074 | 954.8666667 | 2138.8 | 53.3790221 | 1183.933333 | 110.5756453 |
| 1980301 | FKTN | 4.522514851 | 0.022913941 | 0.146665341 | 953.6333333 | 2636.433333 | 331.0946139 | 1682.8 | 154.1642306 |
| 5490603 | LOC401019 | 3.79816115 | 0.021997784 | 0.143638306 | 948.7333333 | 6363.866667 | 259.2323732 | 5415.133333 | 346.3813265 |
| 7050019 | VIM | -57.53779759 | 5.48E-05 | 0.011403989 | 948.1 | 5143.4 | 9.699484522 | 6091.5 | 26.84175851 |
| 4890433 | LOC729679 | 8.880195844 | 0.003889689 | 0.058461487 | 946.3333333 | 2274.066667 | 76.99703457 | 1327.733333 | 167.7523572 |
| 5360347 | NQO1 | 11.77971871 | 3.76E-04 | 0.022553918 | 943.0333333 | 2419.033333 | 87.26650751 | 1476 | 107.7556959 |
| 6900669 | PDE4C | 3.560947452 | 0.049835779 | 0.234015272 | 942.5 | 2235.233333 | 430.8829346 | 1292.733333 | 156.5287301 |
| 4250392 | LOC283412 | 5.666803535 | 0.020991959 | 0.140108837 | 941.1333333 | 4166.166667 | 78.70605652 | 3225.033333 | 276.6792427 |
| 5090671 | GDF15 | 42.8979403 | 1.04E-05 | 0.007702029 | 939.4333333 | 1613.233333 | 32.18529685 | 673.8 | 20.07087442 |
| 7000435 | LOC731365 | -1.949027337 | 0.179586519 | 0.485318515 | 938.2333333 | 4916.966667 | 176.6538517 | 5855.2 | 814.8553062 |
| 1500538 | LOC645138 | 7.380723326 | 0.00330876 | 0.054226066 | 937.4666667 | 2136.9 | 118.5113075 | 1199.433333 | 185.3481139 |
| 2850402 | PFN1 | -3.543873614 | 0.043378972 | 0.215139694 | 937.3666667 | 2925.966667 | 187.302278 | 3863.333333 | 418.0957107 |
| 6380441 | ENO1 | -3.781747305 | 0.054604808 | 0.246641709 | 930.8666667 | 7041.033333 | 92.396663 | 7971.9 | 416.2069798 |
| 4210088 | CKS1B | -4.453509442 | 0.033994119 | 0.18641606 | 929.8666667 | 1679.666667 | 105.7147262 | 2609.533333 | 345.8458809 |
| 5420398 | TRMT112 | 8.339418452 | 0.004641953 | 0.063679397 | 928.9 | 3213.133333 | 80.28463946 | 2284.233333 | 175.4290265 |
| 3370703 | FEN1 | -12.95256211 | 0.004007175 | 0.059300873 | 927.7333333 | 885 | 27.47871904 | 1812.733333 | 120.9774497 |
| 4060333 | VEGFB | 14.74754156 | 5.52E-04 | 0.025451648 | 926.7666667 | 2403.366667 | 95.30720504 | 1476.6 | 52.57309198 |
| 3420368 | LOC644464 | -3.065958774 | 0.046253316 | 0.223400304 | 923.9333333 | 7627.9 | 281.9868259 | 8551.833333 | 439.229807 |
| 2320382 | LOC100130445 | 4.175146477 | 0.049296347 | 0.232306123 | 922.3 | 2145 | 378.7510132 | 1222.7 | 54.23393771 |
| 840647 | RPL36 | 8.011452802 | 0.008831522 | 0.087725114 | 919.6666667 | 3751.366667 | 59.52960048 | 2831.7 | 189.7082233 |
| 1580537 | PCBP2 | 6.91795948 | 0.019978291 | 0.136027687 | 917.4 | 3281.233333 | 11.89341555 | 2363.833333 | 229.3814799 |
| 4570091 | NDUFAF3 | 18.69615642 | 3.10E-04 | 0.021054803 | 916.9333333 | 1820.566667 | 75.10142032 | 903.6333333 | 39.69512984 |
| 5810440 | HNRNPAB | -6.041937718 | 0.009706971 | 0.091708389 | 916.2666667 | 2039.933333 | 116.9528252 | 2956.2 | 235.1940263 |
| 3120196 | ALDH3A2 | 34.83290818 | 9.28E-06 | 0.007693729 | 915.2 | 1445.3 | 26.90650479 | 530.1 | 36.70163484 |
| 1980309 | IL8 | -14.34137903 | 0.004412603 | 0.062133689 | 914.3 | 3168.566667 | 11.60014368 | 2254.266667 | 109.8117177 |
| 6770309 | MYL6 | -5.914626351 | 0.009638134 | 0.091404829 | 913.2333333 | 2103.1 | 237.449342 | 3016.333333 | 123.0375688 |
| 360689 | HEBP1 | 54.82988146 | 7.56E-06 | 0.007693729 | 913.1 | 1616.9 | 14.33736377 | 703.8 | 25.02878343 |
| 1400673 | CDCA5 | -22.97256568 | 0.00141541 | 0.036517782 | 908.9333333 | 555.8 | 11.56589815 | 1464.733333 | 67.54734142 |
| 6450646 | HNRPM | -16.93733004 | 6.06E-04 | 0.026421547 | 908.2 | 1339.2 | 39.5599545 | 2247.4 | 84.02803104 |
| 7100632 | C17ORF45 | 3.951612009 | 0.016874152 | 0.123112357 | 900.3333333 | 4171.6 | 272.0923924 | 3271.266667 | 285.8290631 |
| 4590576 | LOC286444 | 5.374674213 | 0.009759118 | 0.092086928 | 899.1 | 4941.6 | 247.3015164 | 4042.5 | 150.9779123 |
| 3440670 | LOC402251 | 4.58946902 | 0.030513439 | 0.174614518 | 897.5 | 2688.933333 | 105.0459582 | 1791.433333 | 322.0128155 |
| 4730086 | SHCBP1 | 3.752102857 | 0.052613652 | 0.241967566 | 896.6 | 2614.7 | 400.526104 | 1718.1 | 104.3241583 |
| 1690577 | RPL9 | -4.067701481 | 0.016683044 | 0.122440808 | 893.4666667 | 7321.133333 | 238.5355389 | 8214.6 | 296.3745603 |
| 2120037 | PIR | 18.54117888 | 8.33E-04 | 0.030052061 | 891.2666667 | 1167.9 | 77.79865037 | 276.6333333 | 29.65507264 |
| 3130220 | TMEM158 | 6.771050838 | 0.008526269 | 0.086180707 | 889.2 | 4634.966667 | 92.38562298 | 3745.766667 | 207.8526 |
| 5270112 | HMGCS1 | 20.15898761 | 0.002050084 | 0.043438272 | 886.9 | 1176.1 | 75.48450172 | 289.2 | 10.43264108 |
| 4060326 | ATP5F1 | -12.90593444 | 0.00536999 | 0.068043844 | 885.5 | 1676.166667 | 118.0472081 | 2561.666667 | 13.69720166 |
| 7570131 | RPS18 | 7.709625368 | 0.003584647 | 0.056225862 | 883.9666667 | 5345.4 | 171.9069225 | 4461.433333 | 99.43371326 |
| 4260044 | SQSTM1 | 3.103275541 | 0.051638763 | 0.239285837 | 880.6666667 | 9776 | 232.6446002 | 8895.333333 | 432.9898998 |
| 4730369 | BTG1 | -10.18185251 | 0.008174871 | 0.084285431 | 879.2 | 1268.766667 | 22.2603534 | 2147.966667 | 147.8962249 |
| 2710240 | UQCRH | -4.94269057 | 0.010397298 | 0.095554723 | 877.8666667 | 6301.133333 | 252.3727666 | 7179 | 175.906026 |
| 830673 | LOC642947 | 3.682872569 | 0.025110877 | 0.154861318 | 877.0666667 | 2986.633333 | 335.3130826 | 2109.566667 | 240.2243604 |
| 6350544 | RPL10A | 6.248243776 | 0.012455002 | 0.104838278 | 876.6 | 5906.833333 | 226.2390181 | 5030.233333 | 88.68102014 |
| 4280017 | FOS | 31.63249507 | 1.95E-05 | 0.008810364 | 875.8666667 | 1190.5 | 39.75235842 | 314.6333333 | 26.82840534 |
| 7050543 | TUBB2B | -11.36099089 | 0.001781056 | 0.040568783 | 875.2333333 | 2023.633333 | 57.68815592 | 2898.866667 | 120.3197961 |
| 1450685 | ZNF430 | 3.839075986 | 0.037271199 | 0.196244105 | 871.6333333 | 2502.933333 | 361.5476502 | 1631.3 | 154.6872975 |
| 1770546 | ATIC | -11.27508349 | 0.001135668 | 0.034040289 | 868 | 2761.833333 | 66.1954933 | 3629.833333 | 115.7485349 |
| 3390128 | TPM2 | -5.166783536 | 0.011423327 | 0.100238 | 865.8 | 1044.5 | 148.4834334 | 1910.3 | 249.3836202 |
| 6100673 | TUBA1C | -4.268821132 | 0.013697135 | 0.110497228 | 865.0333333 | 3324.2 | 227.2707636 | 4189.233333 | 267.4635365 |
| 5220037 | RPS2 | 3.064199055 | 0.03916852 | 0.202228212 | 864.4333333 | 5412.4 | 375.7517398 | 4547.966667 | 312.3530748 |
| 1260162 | DNMT1 | -16.89833787 | 8.52E-05 | 0.013309382 | 863.0333333 | 1665.8 | 57.4223824 | 2528.833333 | 67.28850818 |
| 5720669 | AK2 | -18.48147395 | 0.00187816 | 0.041524891 | 862.2333333 | 1112.633333 | 17.50266646 | 1974.866667 | 78.88867684 |
| 6900458 | LOC728481 | 6.963946145 | 0.00223683 | 0.045227901 | 861.7333333 | 3715.5 | 150.4071474 | 2853.766667 | 152.6892705 |
| 2230296 | ORC6L | 2.543466093 | 0.081720368 | 0.312239969 | 861.3333333 | 7498 | 514.4656354 | 6636.666667 | 281.722245 |
| 6110129 | RPS6P1 | 4.227182008 | 0.018256384 | 0.128973492 | 860.9 | 4307.433333 | 193.3123466 | 3446.533333 | 295.0597284 |
| 2600035 | NCL | -6.386679622 | 0.022631198 | 0.145595999 | 860.0333333 | 1605.066667 | 21.93042027 | 2465.1 | 232.2054909 |
| 650746 | C21ORF55 | 4.490742891 | 0.011166153 | 0.098935459 | 859.7666667 | 2546.9 | 246.2554162 | 1687.133333 | 222.0836404 |
| 7380554 | LSM3 | -4.776822964 | 0.032414606 | 0.180984738 | 858.2333333 | 1104.266667 | 76.00035088 | 1962.5 | 301.7676424 |
| 6200402 | MT1A | -8.253834077 | 0.011301562 | 0.099562776 | 857.2333333 | 452 | 35.79790497 | 1309.233333 | 176.2908487 |
| 5390685 | LOC643433 | 8.303205535 | 0.006812284 | 0.076523364 | 856.4333333 | 2657.9 | 167.9000893 | 1801.466667 | 61.04238964 |
| 360653 | LOC647276 | 2.483012324 | 0.070549363 | 0.286521251 | 855.8666667 | 9434.5 | 462.8639109 | 8578.633333 | 377.0785241 |
| 5090711 | LOC440575 | -1.918200378 | 0.155351515 | 0.447752288 | 854.0666667 | 6744.7 | 696.4029652 | 7598.766667 | 331.2835845 |
| 6180301 | LOC644745 | 1.613344106 | 0.186482891 | 0.495127753 | 851.9666667 | 6099.666667 | 559.0380876 | 5247.7 | 723.9240223 |
| 6040563 | RPS4X | 4.878691533 | 0.023523677 | 0.149168034 | 849.2666667 | 2791 | 106.7956928 | 1941.733333 | 281.9624148 |
| 870019 | LOC729926 | 16.40435939 | 0.001709601 | 0.039898451 | 849.2333333 | 2066.033333 | 85.77554041 | 1216.8 | 26.12604065 |
| 4890487 | B2M | -4.029705423 | 0.015766092 | 0.118726223 | 848.6666667 | 2550.066667 | 254.0016798 | 3398.733333 | 261.808295 |
| 5900373 | LOC389141 | -2.384587648 | 0.125109752 | 0.397731827 | 848 | 3984.433333 | 153.7550758 | 4832.433333 | 596.4476619 |
| 4180278 | LOC440043 | -10.8167807 | 0.002900577 | 0.050847682 | 842.3333333 | 1509.3 | 50.9247484 | 2351.633333 | 124.8967707 |
| 1940709 | LOC645895 | 1.745839663 | 0.195363072 | 0.505847397 | 841.8333333 | 5694.5 | 291.2811185 | 4852.666667 | 782.744111 |
| 5960646 | LOC100130168 | 3.98417239 | 0.041662118 | 0.209784189 | 841.2666667 | 2070.666667 | 347.7131193 | 1229.4 | 113.3636185 |
| 290114 | EBNA1BP2 | -17.46449293 | 3.16E-04 | 0.02108965 | 840.9666667 | 2225.566667 | 40.60472058 | 3066.533333 | 72.85172155 |
| 1470209 | TMCO1 | -13.9442148 | 3.67E-04 | 0.022379094 | 840.9 | 1767.966667 | 87.25584985 | 2608.866667 | 57.41379045 |
| 610437 | CD24 | -48.28860975 | 3.93E-04 | 0.022919732 | 840.1 | 32.36666667 | 2.468467811 | 872.4666667 | 30.03203845 |
| 5420750 | LOC649447 | 10.21087303 | 0.001497779 | 0.037416015 | 838.8 | 3169.866667 | 123.1490696 | 2331.066667 | 71.26747739 |
| 6770176 | ARF4 | -6.216830459 | 0.004554434 | 0.063193103 | 838.5 | 2286.333333 | 137.7102151 | 3124.833333 | 188.7070304 |
| 6280541 | LAMC3 | 17.03349123 | 7.08E-05 | 0.012599133 | 837.8 | 1421.666667 | 58.75902768 | 583.8666667 | 61.68470907 |
| 1500164 | PCBP1 | 5.741496142 | 0.009109802 | 0.088930753 | 837.1666667 | 3282.366667 | 124.158219 | 2445.2 | 219.9233275 |
| 5720520 | SNHG6 | 10.746222 | 0.004426748 | 0.062198666 | 834.7666667 | 3052.5 | 40.38923124 | 2217.733333 | 128.3404197 |
| 6520215 | ANXA1 | -3.996996829 | 0.037444861 | 0.196917435 | 834.4333333 | 2460.433333 | 127.9231931 | 3294.866667 | 338.2073674 |
| 5340154 | LOC643509 | 2.102469684 | 0.103543434 | 0.358327532 | 828.4 | 4730.133333 | 470.1524469 | 3901.733333 | 494.6665274 |
| 2350465 | RPL29 | -8.120690225 | 0.011590678 | 0.100902478 | 822.0333333 | 1609.866667 | 35.54044644 | 2431.9 | 171.6904482 |
| 1980520 | RPL6 | -13.58481647 | 5.87E-04 | 0.026083667 | 821.3 | 9399 | 90.36199422 | 10220.3 | 52.91436478 |
| 5550328 | LOC647302 | -20.94599596 | 9.28E-05 | 0.013760501 | 820.9666667 | 922.1333333 | 37.20085124 | 1743.1 | 56.78653009 |
| 5570494 | MRPL33 | -7.930910119 | 0.01520178 | 0.116422307 | 818.9 | 2402.566667 | 10.85418506 | 3221.466667 | 178.5118857 |

**Supplementary Table 2:** Enriched stemness associated gene sets in TMZ treated GBM43.

|  | PROBE | ID | | RANK IN GENE LIST | RANK METRIC SCORE | RUNNING ES | CORE ENRICHMENT |
| --- | --- | --- | --- | --- | --- | --- | --- |
| 1 | TWIST1 | 1110132 | | 59 | 3.617 | 0.0637 | Yes |
| 2 | THY1 | 6480204 | | 82 | 3.423 | 0.125 | Yes |
| 3 | ITGA4 | 7200768 | | 224 | 2.746 | 0.1706 | Yes |
| 4 | ALDH1A1 | 1070477 | | 555 | 2.150 | 0.1999 | Yes |
| 5 | BMP7 | 1070478 | | 854 | 1.847 | 0.2247 | Yes |
| 6 | ITGA2 | 3890541 | | 898 | 1.804 | 0.2561 | Yes |
| 7 | WWC1 | 1090575 | | 909 | 1.794 | 0.2883 | Yes |
| 8 | ID1 | 580692 | | 1168 | 1.629 | 0.3103 | Yes |
| 9 | DKK1 | 4880609 | | 1423 | 1.490 | 0.3299 | Yes |
| 10 | DACH1 | 1190273 | | 2206 | 1.208 | 0.3292 | Yes |
| 11 | LIN28B | 4220482 | | 2226 | 1.200 | 0.3503 | Yes |
| 12 | CD34 | 4040706 | | 2626 | 1.098 | 0.3587 | Yes |
| 13 | IL8 | 1570553 | | 3072 | 0.998 | 0.3639 | Yes |
| 14 | MAML1 | 1570592 | | 3447 | 0.927 | 0.3698 | Yes |
| 15 | POU5F1 | 770615 | | 4510 | 0.771 | 0.3531 | No |
| 16 | HDAC1 | 3140056 | | 4894 | 0.722 | 0.3551 | No |
| 17 | AXL | 4640403 | | 6068 | 0.603 | 0.3321 | No |
| 18 | CD44 | 360719 | | 7459 | 0.490 | 0.3009 | No |
| 19 | GATA3 | 5910719 | | 8238 | 0.437 | 0.2863 | No |
| 20 | LATS1 | 5340300 | | 8815 | 0.399 | 0.2769 | No |
| 21 | FOXP1 | 20162 | | 10490 | 0.301 | 0.2340 | No |
| 22 | PTPRC | 870095 | | 10839 | 0.282 | 0.2291 | No |
| 23 | CD38 | 2760500 | | 10880 | 0.281 | 0.2330 | No |
| 24 | KLF17 | 2230632 | | 11323 | 0.257 | 0.2249 | No |
| 25 | GSK3B | 6480661 | | 12261 | 0.208 | 0.2016 | No |
| 26 | PTCH1 | 1410289 | | 12319 | 0.205 | 0.2036 | No |
| 27 | YAP1 | 3360768 | | 13869 | 0.131 | 0.1613 | No |
| 28 | WNT1 | 770370 | | 14759 | 0.091 | 0.1372 | No |
| 29 | MYCN | 430204 | | 15697 | 0.048 | 0.1111 | No |
| 30 | SIRT1 | 7000224 | | 16248 | 0.024 | 0.0956 | No |
| 31 | MS4A1 | 1190519 | | 16642 | 0.009 | 0.0844 | No |
| 32 | ITGB1 | 110440 | | 17704 | -0.037 | 0.0545 | No |
| 33 | NANOG | 5890170 | | 20429 | -0.152 | -0.0215 | No |
| 34 | SNAI1 | 5340010 | | 22035 | -0.230 | -0.0636 | No |
| 35 | MYC | 6270646 | | 23356 | -0.294 | -0.0964 | No |
| 36 | TWIST1 | 4490673 | | 24703 | -0.364 | -0.1287 | No |
| 37 | NFKB1 | 7400626 | | 25627 | -0.415 | -0.1479 | No |
| 38 | FGFR2 | 2970008 | | 26120 | -0.444 | -0.1540 | No |
| 39 | ETFA | 4780743 | | 26345 | -0.457 | -0.1522 | No |
| 40 | FOXA2 | 2230086 | | 26689 | -0.478 | -0.1535 | No |
| 41 | ITGA6 | 2140678 | | 27552 | -0.540 | -0.1686 | No |
| 42 | IKBKB | 1260438 | | 27676 | -0.548 | -0.1623 | No |
| 43 | NOTCH1 | 5080167 | | 29110 | -0.669 | -0.1915 | No |
| 44 | PECAM1 | 430747 | | 29287 | -0.689 | -0.1842 | No |
| 45 | PROM1 | 7400452 | | 29504 | -0.709 | -0.1776 | No |
| 46 | EGF | 3360500 | | 31077 | -0.900 | -0.2067 | No |
| 47 | JAK2 | 3940189 | | 31469 | -0.966 | -0.2005 | No |
| 48 | TAZ | 510326 | | 32159 | -1.099 | -0.2005 | No |
| 49 | MERTK | 4730315 | | 32387 | -1.153 | -0.1862 | No |
| 50 | ZEB1 | 1690768 | | 32490 | -1.175 | -0.1679 | No |
| 51 | JAG1 | 5720735 | | 32552 | -1.191 | -0.1482 | No |
| 52 | FZD7 | 460670 | | 33188 | -1.390 | -0.1414 | No |
| 53 | SOX2 | 5080273 | | 33259 | -1.413 | -0.1178 | No |
| 54 | NOTCH2 | 360474 | | 33366 | -1.453 | -0.0946 | No |
| 55 | EPCAM | 1690553 | | 33740 | -1.648 | -0.0756 | No |
| 56 | SAV1 | 2070025 | | 33755 | -1.658 | -0.0460 | No |
| 57 | ATXN1 | 520601 | | 33922 | -1.758 | -0.0190 | No |
| 58 | KLF4 | 510044 | | 34356 | -2.268 | 0.0095 | No |
|  | | |  | | |  |  |
| Dataset | | | TMZ8-- DMSO8.TMZ8-DMSO8.cls.txt#TMZ8_versus_DMSO8 | | |  |  |
| Phenotype | | | TMZ8-DMSO8.cls.txt#TMZ8_versus_DMSO8 | | |  |  |
| Upregulated in class | | | TMZ8 | | |  |  |
| GeneSet | | | CSC | | |  |  |
| Enrichment Score (ES) | | | 0.36984622 | | |  |  |
| Normalized Enrichment Score (NES) | | | 1.3036914 | | |  |  |
| Nominal p-value | | | 0.08829175 | | |  |  |
| FDR q-value | | | 0.08829175 | | |  |  |
| FWER p-Value | | | 0.046 | | |  |  |
